# The Double Framing Effect of Emotive Metaphors in Argumentation

**DOI:** 10.3389/fpsyg.2021.628460

**Published:** 2021-06-14

**Authors:** Francesca Ervas, Maria Grazia Rossi, Amitash Ojha, Bipin Indurkhya

**Affiliations:** ^1^Department of Education, Psychology, Philosophy, University of Cagliari, Cagliari, Italy; ^2^Instituto de Filosofia da Nova (IFILNOVA), Facultade de Ciências Sociais e Humanas, Universidade Nova de Lisboa, Lisbon, Portugal; ^3^Department of Humanities and Social Sciences, Indian Institute of Technology Jammu, Jammu, India; ^4^Department of Cognitive Science, Jagiellonian University, Kraków, Poland

**Keywords:** metaphor, emotions, framing, equivocation fallacy, affective coherence, reasoning, belief in the conclusion, meaning ambiguity

## Abstract

In argumentation, metaphors are often considered as ambiguous or deceptive uses of language leading to fallacies of reasoning. However, they can also provide useful insights into creative argumentation, leading to genuinely new knowledge. Metaphors entail a framing effect that implicitly provides a specific perspective to interpret the world, guiding reasoning and evaluation of arguments. In the same vein, emotions could be in sharp contrast with proper reasoning, but they can also be cognitive processes of affective framing, influencing our reasoning and behavior in different meaningful ways. Thus, a double (metaphorical and affective) framing effect might influence argumentation in the case of emotive metaphors, such as “Poverty is a disease” or “Your boss is a dictator,” where specific “emotive words” (disease, dictator) are used as vehicles. We present and discuss the results of two experimental studies designed to explore the role of emotive metaphors in argumentation. The studies investigated whether and to what extent the detection of a fallacious argument is influenced by the presence of a conventional vs. novel emotive metaphor. Participants evaluated a series of verbal arguments containing either “non-emotive” or “emotive” (positive or negative) metaphors as middle terms that “bridge” the premises of the argument. The results show that the *affective coherence* of the metaphor's vehicle and topic plays a crucial role in participants' reasoning style, leading to global heuristic vs. local analytical interpretive processes in the interplay of the metaphorical and the affective framing effects.

## Introduction

Previous research in argumentation theory showed that reasoning errors, far from just leading to argumentation fallacies, might shed light on how we reason and what influences our evaluation of arguments (Tversky and Kahneman, 1981; Van Eemeren, 1992; Ariely, 2009; Walton, 2010). Reasoning errors have a psychological dimension (Macagno and Walton, 2010; Walton, 2010; Godden, 2015), as they are arguments that seem to be sound without being so in terms of norms and standards (Van Eemeren and Grootendorst, 2004; Tindale, 2006; Walton, 2006). Their psychological dimension is tightly connected to their linguistic dimension, as also the linguistic formulation of arguments may lead to fallacies of reasoning (Oswald et al., 2018, 2020; Hinton, 2019; Schumann et al., 2020). Fallacies of reasoning might reveal how we make sense of arguments, especially when they are formulated in natural languages, where ambiguous, polysemous, and non-literal use of words is widespread (Ervas et al., 2018). Far from being patently irrational when committing a fallacy, people might just be drawn by the intuitive search for alternative reasons, as in the case of metaphors used in argumentation (Ervas et al., 2018). This argumentation style fits more with the “natural logic” invoked in a pragmatic perspective, where the speaker's meaning is grasped as the conclusion of an inference that makes sense of an apparent meaning violation (Grice, 1989).

Metaphors rarely come “alone,” lacking the affective dimension, as they often communicate an emotional meaning (Fainsilber and Ortony, 1987; Schnall, 2005), whether positive as in “My partner is a rose” or negative as in “My job is a jail,” which also contributes to the evaluation of arguments (Macagno and Rossi, 2021). The emotive language used in argumentation might have crucial implications when accepting the conclusion of an argument (Macagno and Walton, 2014; Pollaroli et al., 2019). Here, the term “argumentation” is used in a wider sense, covering all the reasons in support of the conclusions that a speaker wishes the addressee to draw from some premises. Ordinary evaluations might use other sources of reasoning, departing from normative standards and/or independent from the argument itself, i.e., actual premises and their connection to the conclusion, as already shown in the case of arguments featuring metaphors (Ervas et al., 2015, 2018). The paper aims to explore how emotions and figurative language interact in the evaluation of arguments where “emotive metaphors” connect the premises to a conclusion.

In the Western philosophical tradition, the notion of argumentation has often denied both emotions and figurative language the status of reasoning resources. Argumentation has been defined as a critical use of reason in judgment, often in contrast to emotions (Oaksford et al., 1996; Blanchette et al., 2018): rational justification seems to be the unique relevant source of knowledge at a normative level, while emotions are subjective feelings conveying only perspectives. Emotions safeguard compelling and fleeting interests, and can be “recalcitrant” to reason and arguments (Greenspan, 1988, 1992, 2004; Stocker and Hegeman, 1996; DeLancey, 2002, 2007; D'Arms and Jacobson, 2003). Emotions are often processed in an automatic, unconscious, and obliged way, while the critical use of reason is supposed to be conscious and controlled. Previous experimental studies have challenged this view (Blanchette and Caparos, 2013; Blanchette, 2014), and proposed that conscious thought does not always lead to a better performance than unconscious thought when complex decisions have to be made [but see Rossi (2013) and Rossi (2014)]. Due to a wider capacity to deal with multiple information, Dijksterhuis (2004, p. 593) claimed that the “unconscious also actively thinks,” by associating and integrating the various alternatives in memory [see also Dijksterhuis et al. (2006) and Dijksterhuis et al. (2009)]. Other studies have investigated how positive and negative emotions differently influence both content and style of thought, playing an important role in the regulation of the global-local information processing (Fredrickson and Branigan, 2005; Clore and Huntsinger, 2007).

Nonetheless, figurative language has often been considered so rich in suggestion as to be dangerous in argumentation (Beardsley, 1957). Metaphors have been described as the “exemplars of the improper” (Maasen, 2000, p. 199), leading to fallacies of reasoning in argumentation. They have often been counted as semantic anomalies, deviations from the language properly used in argumentation (Hoffman, 1980; Tourangeau and Sternberg, 1982), or as ornamental devices inessential to argumentation. That metaphor is just a deviant or an ornamental use of language has been largely questioned both in philosophy by Max Black (1954, 1962) and in cognitive linguistics by Lakoff and Johnson (1980). In understanding an abstract concept (the *target*) in terms of a concrete concept (the *source*), metaphor implicitly provides a frame to think of and to reason about the target, selecting some relevant properties of the source and neglecting others (Entman, 1993; Burgers et al., 2016). Still, in the conceptual metaphor theory (Lakoff and Johnson, 1980), studies focusing on metaphorical reasoning [see Thibodeau et al. (2019) for a review] widely acknowledge that the metaphorical framing effect can often work covertly and affect reasoning and evaluation of arguments [Thibodeau and Boroditsky, 2011; but see Steen et al. (2014) for criticism].

From this perspective, metaphor plays an alternative role with respect to reasoning, conceived as the critical and deliberate use of rationality. The framing effect is interpreted as a cognitive bias, influencing how people make decisions or express their evaluations based on how an issue or an argument is presented, rather than following proper logical or normative rules. Something similar can be said about affective framing: that it is another strategy to exploit emotions to frame information and manipulate both reasoning and decision-making. Emotions can be used to present the linguistic formulation of an argument with a specific (positive or negative) valence, which can be considered as a special “semantic primitive” determining the intended (positive or negative) meaning (Barrett et al., 2007). Maiese (2014, p. 524) proposed the term “affective framing” to express the idea that emotions are “a spontaneous, non-inferential, and pre-reflective way of discriminating, filtering, and selecting information that allows us to reduce the overwhelming clutter of information” [see also DeLancey (2002), Solomon (2003), and Prinz (2004)]. From this perspective, emotions can strongly influence the evaluation of arguments (e.g., Schwarz and Clore, 1983; Bless et al., 1996; Schwarz and Skurnik, 2003). Scholars have argued for a hidden and overwhelming force of emotions overcoming normative rules in various types of social reasoning (Marcus et al., 2000; Marcus, 2002; MacKuen et al., 2010; Angie et al., 2011). In moral reasoning, scenarios based on strong emotional reactions have been used to insist on the “moral doumbfounding” effect of emotions: for instance, experiencing a strong disgust reaction after reading a story of “consensual incest” brings many participants to remain stubbornly committed to a judgment of moral inappropriateness despite the fact that they are unable to propose adequate arguments to justify their initial emotional intuition [see also Haidt et al. (2000), Haidt (2001, p. 814), and Haidt (2007)].

Previous research has highlighted the evaluative connotations entailed by the framing effect present in metaphors, such as “Poverty is a disease” or “Your boss is a dictator,” where specific “emotive words” (disease/dictator) are intentionally used (Stevenson, 1944; Macagno and Walton, 2014). These examples illustrate how metaphors might guide our reasoning, not only by framing arguments with vividness and forceful figurative images, but also by entailing the communication of emotional attitudes and value judgments (Semino, 2008; Burgers et al., 2016). In this paper, we investigate the double framing effect of emotive metaphors in arguments, and check whether and to what extent the presence of an “emotive metaphor” influences the detection of fallacies.

### The Metaphorical Framing Effect on Argumentation

Scholars have shown that metaphors can be useful in argumentation to introduce a standpoint or to underpin it (Wagemans, 2016, 2019; van Poppel, 2018, 2020), while in the context of science, metaphors have been used to stimulate creative thinking (Blackburn, 1984; Hofstadter, 1995; Indurkhya, 2010). Recent studies have reconsidered traditional approaches to metaphor as a reasoning device (Black, 1962; Hesse, 1963, 1965; Perelman and Olbrechts-Tyteca, 1969; Indurkhya, 2007), claiming that metaphor itself might be considered as an “implicit argument” where the addressee is led along a chain of inferences from the source to the target to draw some conclusion (Santibáñez, 2010; Macagno and Zavatta, 2014; Oswald and Rihs, 2014; Svačinova, 2014). Other studies (Ervas et al., 2018; Ervas, 2019; Cavazzana and Bolognesi, 2020) claimed that metaphors, as implicit arguments, can be considered as *enthymemes*, having a syllogistic form of reasoning with implicit premises. Specifically, the syllogism would have the metaphor as the first premise and the relevant property or properties to attribute to the target as a second premise. Here, the role of the middle term connecting the premises to the conclusion is played by the *vehicle*, the linguistic term that refers to the source concept of the metaphor, which also provides a frame for interpreting the target concept.

When considering the metaphorical framing effect in reasoning, and its influence on the evaluation of arguments, most empirical studies focused on conventional metaphors (Thibodeau and Boroditsky, 2011, 2015), which are quite frequent and already lexicalized for a given linguistic community. On the contrary, novel metaphors might well be used as relevant moves in argumentation (van Poppel, 2018, 2020; Macagno, 2020), which intentionally frame a discourse to interfere with, or even lead, the reasoning process. When argumentation intentionally exploits a metaphor, the robust notion of truth needs to be dropped: sentences featuring a metaphor are literally “patently” false, because of their conventional meaning. Still, from a pragmatic perspective, we find “an alternative truth” in the Gricean natural logic, interpreting the speaker's meaning and making sense of the sentence according to the context (Grice, 1975, 1989; Clark, 1994; Wilson and Sperber, 2002). The Gricean cooperative principle assumes that, beyond being informative, sentences are true. However, sentences featuring conventional and novel metaphors are processed in very different ways when it comes to their truth evaluations. Empirical research (Glucksberg, 2001, 2003; Giora, 2003) has shown that most sentences with conventional metaphors are perceived as true though they are literally false, while most sentences with novel metaphors are processed as false. Response times also suggest a metaphorical interference effect in the truth evaluation of a sentence (Glucksberg et al., 1982). In particular, novel metaphors with unfamiliar meanings cannot be inhibited or ignored, which explains why it takes less time, compared to conventional metaphors, to judge whether sentences featuring novel metaphors are false. Consequently, the process of truth evaluation of the sentence in which the metaphor occurs also influences the evaluation of the whole argument having the metaphorical sentence as a premise.

For conventional metaphors, the relevant properties attributed to the target come from a “system of associated commonplaces” (Black, 1954), which are usually assumed to be true or just believed as belonging to the source concept. The relevant properties are often stereotypically believed to belong to the source concept (Ervas, 2017; Borelli and Cacciari, 2019), possibly leading to fallacies of reasoning (Fischer, 2014, 2015). When they are applied to the target, the preservation of truth in the conclusion is never guaranteed. The speaker's beliefs about the source concept can thus influence the conclusion of the argument featuring a metaphor. People might accept the conclusion of an argument just because they believe in it, rather than because it logically follows from the premises (Ball et al., 2006; Correia, 2011; Ball and Thompson, 2018), thereby influencing the evaluation of the whole argument (Ervas et al., 2018).

### The Affective Framing Effect on Argumentation

As for metaphors, having an embodied (re)framing effect does not automatically mean that emotions can be viewed just as a covert and subconscious force that makes reasoning derails into fallacies. Emotions can have both a bodily and a cognitive-evaluative dimension, as they are the means by which personally relevant environmental information is made available and meaningful to the experiences of a subject (Maiese, 2015). Therefore, precisely for their framing effect, emotions can be considered as an important source of knowledge (Damasio, 1994). As cognitive processes used to represent the positive and negative valence of objects, people and/or actions in the world, they might play a crucial role in reasoning because of their strong evaluative dimension (Caruana and Cuccio, 2017). Although emotions are not explicitly intentional (Damasio, 1994; LeDoux, 1996), they can guide our behavior and be useful predictors of actions: negatively valenced stimuli represent potential threats demanding an immediate response (Rozin and Royzman, 2001; Citron et al., 2014). Previous research showed that positively valenced contexts reduces or even eliminates possible framing effects in decision-making (Cassotti et al., 2012).

Some studies have shown that when emotions are conveyed by verbal stimuli, they strongly affect reading times (Kissler et al., 2006; Citron, 2012). Specifically, emotionally-valenced terms are processed faster and more accurately than neutral terms (Larsen et al., 2006; Kousta et al., 2009). Behavioral ratings of emotionally-valenced stimuli show that both highly positively valenced and negatively valenced stimuli are more arousing than neutral stimuli (Bradley and Lang, 1999) and negatively valenced stimuli are usually rated as more arousing than positively valenced stimuli (Citron et al., 2013). When emotions are more difficult to translate into plain language, we might resort to non-literal and/or figurative language, where meanings can afford one with the liberty to implicitly convey emotions without being overtly committed to the literal value of the words (Gibbs et al., 2002; Ervas, 2020). Kövecses (2000, 2005), who dedicated his work to the different perspectives entailed by metaphors expressing emotions, refers to this use of language as the figurative descriptive function of emotive terms.

Previous research investigated how frames affect individuals' beliefs on a variety of issues, focusing on the place of emotions in the framing process (Petty and Cacioppo, 1986a; Scherer et al., 2014). Being spontaneous action tendencies, emotions can have a direct behavioral effect, but can also indirectly affect judgment, by generating other emotions, or by changing the preference ordering in motivations (Elster, 1999). Previous studies also focused on different cognitive processes explaining how framing effects operate, such as *accessibility change*, making information more salient, *belief importance change*, altering the weight of information, or *belief content change*, adding new information potentially changing the conclusion (Slothuus, 2008), but also affective factors might have a role in mediating framing effects (Gross and D'Ambrosio, 2004; Gross, 2008). The valence of the terms in a message modulates the subjective state of feeling pleasure or displeasure in response to the message (Lecheler et al., 2013). Frames themselves often rely upon emotional appeals, represented by “emotion-laden” terms, which could be properly designed to elicit positive vs. negative emotional reactions. This is also the case of emotive metaphors, whose framing effect does not merely depend on the properties selected from the source term, the *vehicle*, but also on the affective (positive vs. negative) valence of the target term, the *topic*. Both the metaphorical and the affective framing play a role in the interpretation of the target, influencing people's evaluation of the whole sentence featuring the metaphor. However, it is not clear how the metaphorical framing and the affective framing interact in the evaluation of an argument featuring an emotive metaphor.

### Current Research

In argumentation theory, the fallacies of ambiguity are based on some equivocation of meaning, possibly caused by different literal meanings of the same word (Walton, 1996; Tindale, 2006). In linguistics, lexical ambiguity includes both homonymy (referring to words with two completely different literal meanings) and polysemy (referring to words with two partially overlapping literal meanings). The vehicle of a conventional metaphor has a literal and a lexicalised non-literal meaning and can be considered more similar to polysemy (Carston, 1997, 2002). Novel metaphors cannot be considered as cases of lexical ambiguity, because of the completely new and creative non-literal meaning of their vehicles (Ervas, 2015). However, fallacies of ambiguity can also be caused by metaphors, whose vehicle can have both a literal and a non-literal meaning. Previous research (Ervas et al., 2015, 2018) investigated the role of different types of meaning ambiguity as a possible source of fallacious reasoning in argumentation, ranging from literal (homonymy and polysemy) to metaphorical (conventional and novel) words. Ervas et al. (2018) showed that people commit an ambiguity fallacy, especially when evaluating syllogisms with a conventional metaphor as the middle term, i.e., the term that connects the premises of an argument, and with a plausible conclusion. The authors suggested that, when arguments do not present a patently false conclusion, the participants could accept the conclusion just because it is believed to be true on the basis of *a priori* beliefs, and not because it logically follows from the premises. The *belief in the conclusion bias* is well-known to influence the overall evaluation of arguments (Evans et al., 1983; Oakhill et al., 1989; Oakhill and Garnham, 1993; Ball et al., 2006; Correia, 2011; Ball and Thompson, 2018), possibly leading to a reinterpretation of the premises in a creative search for alternative reasons to hold the conclusion (Oakhill et al., 1989; Oakhill and Garnham, 1993; Ball et al., 2006). For conventional metaphors, which go unnoticed to the participants in reading the premises, the meaning of the middle term could be revitalized to justify the conclusion (Ervas et al., 2018).

An example of a standard *equivocation fallacy* (or *quaternio terminorum*) featuring a metaphor as middle term is the following:

P1: B.B. King is a *myth*
P2: A *myth* is a traditional story
C: B.B. King is a traditional story

In the first premise (P1), “myth” is the middle term having a metaphorical meaning, i.e., famous, outstanding person, while in the second premise (P2), “myth” is the middle term having a literal meaning, i.e., traditional story. Because of the meaning shift of the term “myth,” the argument assumes the structure of a *quaternio terminorum* (Barth, 1974; Macagno and Walton, 2009), i.e., a fallacious argument based on the ambiguity of its middle term (Hamblin, 1970; Woods and Walton, 1989; Copi et al., 2014). If the middle term assumes a different meaning in the two premises, then the syllogism contains four terms, rather than three, which causes the fallacy. We called “metaphoric fallacy” a *quaternio terminorum* based on an ambiguity connected to the metaphorical premise of the argument (Walton, 1996; Lightbody and Berman, 2010; Fischer, 2014).

As far as we know, previous empirical research on argument evaluation did not include “emotive metaphors” in the premises and did not focus on their double framing effect on the acceptance of the conclusion. The double framing effect of metaphors might have a strong influence on people's beliefs involved in the argument's evaluation process, possibly leading to equivocation fallacies. The double framing effect of metaphors might depend both on the type (conventional vs. novel) and on the emotional meaning (positive vs. negative valence) of the metaphor featured in the first premise of the argument. This raises the following research questions:

Q1. Under what conditions does the double framing effect of emotive metaphors mostly influence the evaluation of the argument, leading to a fallacy of equivocation?
Q2. Under what conditions does the metaphorical framing influence the evaluation of an argument? Are participants more prone to commit a fallacy of equivocation in the case of conventional metaphors or in the case of novel metaphors?
Q3. Under what conditions does the affective framing influence the evaluation of the argument? Are participants more prone to commit a fallacy of equivocation in the case of negatively valenced metaphors or in the case of positively valenced metaphors?

Two empirical studies were designed to address these questions and to investigate the double framing effect of emotive metaphors in arguments' evaluation. The studies were approved by the Ethics Committee of the Department of Education, Psychology, Philosophy, University of Cagliari (n. 25, 10/07/2018). We tested participants' evaluation of arguments having the standard syllogistic form, with two premises and a plausible conclusion. An example of syllogism with an emotive metaphor is given below:

P1: Freedom is a smile
P2: A smile is an expression of joy
C: Freedom is an expression of joy

The middle terms, such as *smile*, bridge the premises and have their metaphorical meaning in the first premise, diverging from the literal meaning in the second premise, thus leading to a fallacious - but still meaningful - conclusion. The middle terms in the first premise might be either the vehicle of a *conventional metaphor* (CM), or the vehicle of a *novel metaphor* (NM), as in the example provided above. The middle terms might be positively valenced (+), as in the example, negatively valenced (−), or non-emotive (0), based on their emotional meaning. We also devised two sets of first premises having the valence of the metaphor vehicle respectively *coherent* with the valence of the topic (Experiment 1) and *incoherent* with the valence of the topic (Experiment 2), to check whether the double framing effect depends on the emotional meaning of the vehicle or on the emotional meaning of the overall premise featuring the metaphor.

### Hypotheses and Expectations

Based on previous research, we advanced the following hypotheses:

H1: Emotive metaphors, i.e., metaphors based on an “emotive word” as a vehicle, entail a double framing effect in argumentation, systematically leading one to commit an equivocation fallacy.

As only the metaphor vehicle is the middle term that bridges the premises of the syllogism, we expected the participants to provide more inaccurate responses to syllogisms with emotive metaphors than to syllogisms with non-emotive metaphors, independently from the affective coherence of the vehicle and the topic.

H2: The metaphorical framing effect on the evaluation of arguments is stronger in the case of conventional rather than novel metaphors.

We therefore expected the participants to commit more equivocation fallacies and provide less accurate responses for arguments that contain conventional metaphors compared to novel metaphors, because in the former case participants are not aware of the metaphorical framing effect, while in the latter case they are aware of the “deviant,” creative use of language in the premises leading to the conclusion.

H3: The affective framing effect is stronger especially in the case of negatively valenced metaphors when compared to non-emotive metaphors.

We therefore expected a lower accuracy in the case of arguments with negatively valenced metaphors compared to non-emotive metaphors, as negative stimuli represent potential threats demanding an immediate rather than a deliberative response.

We also aimed to explore why participants could accept a fallacious syllogism as sound, when the middle term is an emotive metaphor and the argument conclusion is plausible. Based on previous research (Ervas et al., 2018), we were interested to check whether the reasons could be:

1. *Understandability*: the overall syllogism with the emotive metaphor is simply not understood by the participants, leading to an inaccurate evaluation of the argument;
2. *Convincingness*: the participants feel convinced by the argument, thus thinking it is also sound;
3. *Emotional appeal*: the participants is emotionally engaged or persuaded by the argument;
4. *Logical relation*: the participants think to have found a logical connection between the premises and the conclusion of the argument, when it is not the case;
5. *Ambiguity*: the participants think that in the argument there is no word used in two different meanings, when it is instead the case;
6. *Belief in the conclusion*: the participants believe in what is stated in the conclusion, independently of the content of the premises;
7. *Real-world experience*: the participants are used to hearing similar arguments in their everyday experience and thus uncritically accept them.

We expected that the participants' reasons to accept the fallacious arguments as sound differ from the reasons to reject them as fallacious. For instance, finding an ambiguity could be a reason to reject an argument as fallacious, while the emotional appeal of an argument could be a reason to accept a fallacious argument as sound. We also expected some differences on the participant's evaluation according to the syllogism type: for instance, syllogisms with non-emotive metaphors would be less emotionally appealing than syllogisms with emotive metaphors, or the participants' belief in the conclusion would be higher in the case of syllogisms with conventional metaphors compared to novel metaphors (Ervas et al., 2018).

### Method

For both the experiments on syllogisms with affectively coherent and incoherent metaphors, we presented the participants a series of fallacious syllogisms with either an emotive or a non-emotive metaphor as middle term and with a plausible conclusion, asking whether the conclusion of the syllogisms follows from the premises (“Yes”/“No” answer). Literal syllogisms were planned as fillers to check participants' basic ability to distinguish between clearly strong (SL) and weak (WL) literal arguments, without any explicit instruction, and to understand whether participants are prone to accept fallacious literal arguments with plausible conclusions (PL). We planned to explore why participants answered “Yes” vs. “No,” asking the participants to rate the syllogisms on a 1–5 Likert scale (1 = least likely; 5 = most likely), based on the following measures:

1. *Understandability*: Do you understand the argument?
2. *Convincingness*: Is the argument convincing in any way?
3. *Emotional appeal*: Is the argument emotionally appealing?
4. *Logical relation*: Is the conclusion logically related to the premises?
5. *Ambiguity*: Is the ambiguity at any level influencing?
6. *Belief in the conclusion*: Do you believe in C (independent of P1 and P2)?
7. *Real-world experience*: Do you have any experience of similar arguments?

#### Experimental Design

Two experiments were designed to test both the metaphorical and the affective framing effects, and their interaction effect on the evaluation of the metaphoric fallacy. For this, we planned a 3 × 2 experimental design, having 3 “affective framing” conditions (non-emotive vs. emotive, i.e., positively valenced vs. negatively valenced metaphors) × 2 “metaphorical framing” conditions (conventional vs. novel metaphors). The experiments 1 and 2 were designed to test respectively affectively coherent vs. affectively incoherent metaphors as first premises of the syllogisms.

#### Data Analyses

We planned the following coding and data analyses for both the experiments. In the case of fallacious syllogisms with metaphors, we calculated the scores for accuracy in the following way: 0 for the incorrect answers “yes,” 1 for the correct answers “no.” Indeed, participants answering “yes” think that the conclusion follows from the premises, thus accepting the fallacious arguments as sound, even though the middle term is used with different meanings in the premises. For the same reasons, in both the case of clearly weak literal arguments and weak literal arguments with plausible conclusions, we attributed 0 for the incorrect answers “yes,” 1 for the correct answers “no.” On the contrary, in the case of strong literal arguments with middle terms used with the same meaning in both the premises, we assigned 1 to the correct answers “yes” and 0 for the incorrect answers “no.”

We checked the accuracy of responses to the literal fillers, to ensure that the participants implicitly understood how to distinguish between strong and weak syllogisms, even in case of plausible conclusions. A Chi-squared test determined whether a significant difference existed between the participants' answers and the correct answers in the case of literal fillers. A series of paired *t*-tests were used to see whether weak literal (WL) arguments, especially when having plausible conclusions (PL), were more difficult to detect compared to strong literal (SL) arguments. A Chi-squared test was also used to check whether a significant difference existed between the participants' answers and the correct answers in the case of arguments with metaphors, to see whether participants systematically failed to detect the fallacy in each condition. We calculated the effect sizes, reporting the Phi coefficient (φ) (small effect size: φ = 0.1; medium effect size: φ = 0.3; large effect size: φ = 0.5).

Finally, we performed two statistical analyses for both Experiments 1 and 2:

1. An analysis of variance (ANOVA) for accuracy to assess the main effects of the two factors, metaphorical and affective middle term type, and the interaction of the two factors on the evaluation of the arguments. We calculated the effect sizes, reporting the Eta squared coefficient (η^2^) (small effect size: η^2^ = 0.0099; medium effect size: η^2^ = 0.0588; large effect size: η^2^ = 0.1379);
2. An exploratory analysis on the seven measures for both committing the fallacy (accepting the conclusion as following from the premises, incorrect answers “yes”) and detecting the fallacy (discarding the conclusion as not following from the premises, correct answers “no”). A multiple linear regression was planned to explore the impact of the seven measures (Understandability, Convincingness, Emotional appeal, Logical relation, Ambiguity, Belief in the conclusion, and Real-world experience) on participants' evaluation of the different types of syllogisms, assuming that they could have different reasons to accept the fallacious syllogisms as sound (“yes” answer) and to discard the fallacious syllogisms as actually fallacious (“no” answer). We planned to create separate linear models for 3 “affective framing” conditions (non-emotive/positive/negative) × 2 “metaphorical framing” conditions (conventional/novel) for “yes” and “no” responses. A multiple linear regression was calculated to predict the dichotomous dependent variable (“yes”/“no”) based on the seven predictors (understandability, convincingness, emotional appeal, logical relation, ambiguity, belief in the conclusion, and real world experience) for novel and conventional metaphors, by entering all the predictors simultaneously.

#### Rating and Pilot Studies

To provide the materials for both the experiments, we pre-tested the (1) *vehicles* of the metaphors, (2) metaphors in the first premises of the syllogisms, and (3) separate premises and conclusions of the syllogisms in a series of rating studies (*N* = 257 participants).

##### Metaphors' Vehicles

We selected a set of terms (*N* = 206 nouns, GRADIT; De Mauro, 2000) that could be used to form the first premises of the arguments. All the terms were preselected according to their number of letters (CM: M = 6.92, SD = 1.24; NM: M = 6.92, SD = 0.67) and frequency (both CM and NM vehicles belonging to the “common terms” frequency category in the GRADIT, De Mauro, 2000). From this set, we selected the *metaphors' vehicles*, which were the *same* in both Experiment 1 and 2, constituting the middle terms of the syllogisms. We selected the *metaphors' vehicles*, based on the results of a rating study on their familiarity and emotional (positively and negatively valenced) meaning, on a 1–5 Likert Scale (1 = very negative/very unfamiliar, 5 = very positive/very familiar). Terms with insufficient familiarity (*M*_familiar_ < 2) were excluded. We used three sets of *metaphors' vehicles* to form the metaphors: (1) terms with definite emotional meanings (*M*_positive_ < 4; *M*_negative_ > 2) were used as vehicles of non-emotive metaphors; (2) terms with definite positive emotional meanings (*M*_positive_ > 4) were used as vehicles of positively valenced metaphors, and (3) terms with definite negative emotional meanings (*M*_negative_ < 2) were used as vehicles of negatively valenced metaphors. Terms already having a lexicalized metaphorical meaning in the GRADIT were used as vehicles of *conventional metaphors*. Unambiguous terms were used as vehicles of *novel metaphors*, ensuring that they had no already lexicalized figurative meanings in the GRADIT.

##### Metaphors

From the preselected set of terms, we also selected the *metaphors' topics*, based on their ratings for familiarity and emotional (positively and negatively valenced) meaning, on a 1–5 Likert Scale (1 = very negative/unfamiliar, 5 = very positive/familiar). We devised three sets of *metaphors' topics* to form the metaphors: (1) non-emotive topics with definite emotional meanings (*M*_positive_ < 4; *M*_negative_ > 2); (2) positively valenced topics with definite emotional meanings (*M*_positive_ > 4); (3) negatively valenced topics with definite emotional meanings (M_negative_ < 2). A set of conventional and novel *non-emotive metaphors*, the same for both the experiments, was generated with non-emotive metaphors' vehicles and topics for the first premises of the syllogisms with non-emotive metaphors. Two sets of *emotive metaphors* constituted the first premises of the syllogisms with emotive metaphors: (1) a set of first premises where the emotional meaning of the metaphors' vehicles was coherent with the emotional meaning of the metaphors' topics (Experiment 1); (2) a set of first premises where the emotional meaning of the metaphors' vehicles was incoherent with the emotional meaning of the metaphors' topics (Experiment 2).

In two separate rating studies for Experiments 1 and 2, the sets of metaphors were tested along some major psycholinguistic variables (Bambini et al., 2014): emotional (positively and negatively valenced) meaning, familiarity, meaningfulness (i.e., confidence in metaphor interpretation), and comprehension difficulty using a 1–5 Likert scale (1 = very negative/unfamiliar/meaningless/easy, 5 = very positive/familiar/meaningful/difficult). Metaphors with insufficient meaningfulness (*M*_meaningful_ < 2) and metaphors too difficult to understand (*M*_difficult_ > 4) were excluded. We deemed metaphors with no definite emotional meanings (*M*_positive_ < 4; *M*_negative_ > 2) as non-emotive metaphors, and the metaphors with definite emotional meanings (*M*_positive_ > 4; *M*_negative_ < 2) as positively valenced and negatively valenced emotive metaphors (see Table A1 in Appendix for M and SD for each measure).

##### Premises and Conclusions

The second premises of the syllogisms, the *same* in both materials of Experiment 1 and 2, made explicit the literal meaning of the middle term provided in the GRADIT, which differs from the metaphorical meaning of the middle term in the first premises. We pre-tested the separate premises of all the arguments to ensure that the participants actually attributed different meanings to the middle terms in the first and the second premises of the syllogisms. Besides the topics of emotive metaphors in the first premises, the materials of the experiments differed for their conclusions, which connected the metaphor's topic to the last term of the second premises. We also separately tested all the premises and conclusions of the syllogisms to ensure that participants perceived them as true or at least plausible, to avoid false premises leading to an “ex falso quodlibet.”

##### Pilot Study

A pilot study on syllogisms with emotionally coherent premises showed that participants (*N* = 13, nine women, four men) accepted more fallacious arguments as sound when having emotive metaphors rather than non-emotive metaphors as middle terms.

### Experiment 1

The goal of the experiment was to test the evaluation of metaphoric fallacies, having a syllogistic form and an emotive metaphor (with coherently-valenced vehicle and topic) in the first premise. We aimed to understand whether and why participants were prone to accept a *quaternio terminorum* with a plausible conclusion as sound, especially in the case of emotive metaphors. We also aimed to explore how different factors (understandability, convincingness, emotional appeal, logical relation, ambiguity, belief in the conclusion, and real-world experience) contribute to the participants' evaluation of arguments with a plausible conclusion, comparing emotive, and non-emotive metaphorical middle terms conditions.

#### Participants

The participants (93 adults, 50 women, 43 men) were undergraduate students in Communication Science at the University of Cagliari, had Italian as their first language, and normal/corrected vision. Since we aimed to check for the participants' intuitive answers on different measures concerning the acceptability of argument conclusions, we excluded participants (*N* = 2) who had advanced training in logic and/or argumentation theory, resulting in 91 participants (48 women, 43 men, *M*_age_ = 23.58 years, *SD*_age_ = 7.41 years).

#### Materials

Participants were presented with a set of *N* = 36 arguments in Italian (see Table A2 in Appendix), having the structure of syllogisms with plausible conclusions. The set of arguments contained *N* = 8 non-emotive metaphors, four conventional (*CM0*) and four novel (*NM0*); *N* = 8 positively valenced metaphors, four conventional (*CM*+) and four novel (*NM*+); *N* = 8 negatively valenced metaphors, four conventional (*CM*−) and four novel (*NM-*), in their first premise. Table 1 presents an example of argument translated into English for each middle term condition.

**Table 1:** Examples of arguments in English for each middle term condition (Experiment 1).

	**Non-emotive metaphors (0)**	**Positively valenced metaphors (+)**	**Negatively valenced metaphors (-)**
CM	[P1] That girl is a *gem*. (*gemma*) [P2] A *gem* is a precious stone. [C] That girl is a precious stone.	[P1] Peace is a *thaw*. (*schiarita*) [P2] A thaw is the defreezing of ice. [C] Peace is the defreezing of ice.	[P1] An insult is a *scar*. (*sfregio*) [P2] A *scar* is a wound. [C] An insult is a wound.
NM	[P1] The rooster is a *pharaoh*. (*faraone*) [P2] A *pharaoh* is a leader. [C] The rooster is a leader.	[P1] Freedom is a *smile*. (*risata*) [P2] A *smile* is an expression of joy. [C] Freedom is an expression of joy.	[P1] A betrayal is a *scald*. (*ustione*) [P2] A *scald* is a serious burn. [C] A betrayal is a serious burn.

The set of arguments also included 12 literal arguments as fillers: four clearly weak literal arguments, four clearly strong literal arguments, and four literal arguments with plausible conclusions (see Table A3 in Appendix for the literal arguments in Italian).

#### Procedure

The data was collected through an online form. After the participants signed the informed consent, the form gathered information about gender, age, language, and education. Participants were then asked to read the instructions and complete two practice trials to familiarize themselves with the task. The syllogisms were then randomly presented, followed by some questions. After each argument, the following question appeared: “Does the conclusion follow from the premises?,” asking the participants to answer “Yes” or “No.” Participants were then asked to rate the arguments on a 1–5 Likert scale (1 = least likely, 5 = most likely), answering the questions for the measures: (1) Understandability; (2) Convincingness; (3) Emotional appeal; (4) Logical relation; (5) Ambiguity; (6) Belief in the conclusion; (7) Real-world experience. The experiment lasted 30 min ca.

#### Results

All the data collected are available at the following **OSF address** https://osf.io/jzpva/?view_only=8edc3b523cbb4afba4bd71978d847a48. Mean (M) and standard deviation (SD) for accuracy are presented in Table 2. First of all, we checked whether the participants correctly answered in the case of literal arguments, to understand whether they carefully performed the task and whether they have a basic ability in detecting clearly strong and clearly weak arguments. The participants performed almost at ceiling for all literal arguments. The Chi-squared test showed that the participants provided significantly more correct than incorrect answers to literal arguments (*p* < 0.001; LS: χ^2^ = 134.00, φ = 0.86; LW: χ^2^ = 102.34, φ = 0.75; LP: χ^2^ = 57.36, φ = 0.56). A series of paired *t*-tests suggested that fallacious literal arguments with plausible conclusions were more difficult to detect when compared to both clearly strong (*p* < 0.001) and clearly weak arguments (*p* < 0.01).

**Table 2:** Mean (M) and standard deviation (SD) values of correct answers for each middle term condition (Experiment 1).

	**Positively valenced**		**Negatively valenced**		**Non-emotive**	
	***M***	***SD***	***M***	***SD***	***M***	***SD***
CM	0.16	0.21	0.09	0.17	0.15	0.25
NM	0.23	0.29	0.21	0.27	0.31	0.30
**Literal**	**Strong arguments**		**Weak arguments**		**Arguments with plausible conclusion**	
	0.97	0.13	0.90	0.24	0.79	0.26

The Chi-squared test showed that participants provided more incorrect than correct answers to arguments with metaphors (*p* < 0.001; CM0: χ^2^ = 76.43, φ = 0.65; CM+: χ^2^ = 69.24, φ = 0.62; CM−: χ^2^ = 99.98, φ = 0.74; NM0: χ^2^ = 28.45, φ = 0.40; NM+: χ^2^ = 51.65, φ = 0.53; NM-: χ^2^ = 51.86, φ = 0.53). A two-way ANOVA was performed for accuracy to assess the main effects of the metaphorical and affective middle term type and the interaction of the two factors on the evaluation of the fallacious arguments. The results of the main effects are reported in Table 3.

**Table 3:** Main effects of metaphorical and affective middle term type for accuracy in Experiments 1 and 2.

	**Accuracy (Experiment 1)**						**Accuracy (Experiment 2)**					
	**SS**	**df**	***F***	***P***	**η^**2**^**	***f***	**SS**	**df**	***F***	***p***	**η^**2**^**	***f***
C (Metaphorical)	1.9	1	29.30	<0.001	0.05	0.23	0.0	1	0.01	0.91	0.00	0.08
C (Affective)	0.6	2	4.63	0.01	0.016	0.13	5.6	2	2.11	0.12	0.007	0.01
C (Metaphorical): C(Affective)	0.2	2	1.65	0.192	0.006	0.08	20.9	2	7.89	<0.001	0.027	0.17

*SS, Sum of squares; df, degrees of freedom; η^2^, Eta squared (small: η^2^ = 0.01; medium: η^2^ = 0.059; large: η^2^ = 0.138); f, Cohen's effect size (small: f = 0.1, medium: f = 0.25, large: f = 0.4)*.

Overall, the results showed a significant main effect of the metaphorical type [*F*_(1,89)_ = 29.30; *p* < 0.001; η^2^ = 0.05] and the affective type [*F*_(2,88)_ = 4.63; *p* = 0.01; η^2^ = 0.016], but no significant interaction of the metaphorical type and the affective type [*F*_(2,86)_ = 1.65; *p* = 0.192; η^2^ = 0.006] on participant's evaluation of the arguments. The significant main effect of the metaphorical type is due to the lower number of correct answers in the case of arguments with conventional metaphors when compared to arguments with novel metaphors. A *post-hoc* Tukey's test, corrected for multiple comparisons, was performed to determine the statistical significance of the difference between specific affective conditions (see Table 4 for all the results). The *post-hoc* analysis revealed that the significant main effect of the affective type is due to the lower number of correct answers in the case of arguments with negatively valenced metaphors when compared to arguments with non-emotive metaphors [*t*_(89)_ = 0.32; *p* < 0.01].

**Table 4:** *t*/*p*-values for accuracy, comparing middle term affective conditions (Experiment 1).

	**Comparisons**	***t***	***p***
C (Affective)	neutral/negative	0.32	<0.01
	positive/negative	0.18	0.19
	positive/neutral	−0.13	0.40

We analyzed the answers provided by the participants on different possible factors influencing the arguments' evaluation (see M and SD values for each measure in Table 5). A multiple linear regression analysis was performed on the data: separate linear models were created for 3 “affective framing” conditions × 2 “metaphorical framing” conditions for both “yes” and “no” responses. Table 6 presents the results of the linear regression for all middle term conditions.

**Table 5:** Mean (M) and standard deviation (SD) values of each predictor for each middle term condition (Experiment 1).

**Predictor**	**CM+**		**CM-**		**CM0**		**NM+**		**NM-**		**NM0**	
	***M***	***SD***	***M***	***SD***	***M***	***SD***	***M***	***SD***	***M***	***SD***	***M***	***SD***
Understandability	3.23	0.66	3.57	0.66	3.21	0.67	2.92	0.80	3.00	0.67	2.51	0.67
Convincingness	3.01	0.65	3.38	0.70	2.99	0.77	2.63	0.85	2.64	0.72	2.27	0.84
Emotional appeal	2.33	0.82	2.50	0.80	2.34	0.78	2.49	0.99	2.30	0.79	1.49	0.55
Logical relation	3.11	0.75	3.45	0.71	3.24	0.81	2.89	0.84	2.92	0.78	2.55	0.76
Ambiguity	2.27	0.75	1.89	0.63	2.25	0.80	2.46	0.91	2.52	0.78	2.84	0.89
Belief in the conclusion	3.18	0.67	3.48	0.76	3.11	0.77	2.81	0.92	2.94	0.86	2.56	0.77
Real world experience	2.85	0.82	3.20	0.86	2.93	0.89	2.75	0.99	2.84	0.85	1.96	0.83

*Positively valenced (CM+), negatively valenced (CM-), non-emotive (CM0) conventional metaphor. Positively valenced (NM+), negatively valenced (NM-), non-emotive (NM0) novel metaphor*.

**Table 6:** *t/p*-values for each predictor in the evaluation of arguments, comparing the middle term conditions (Experiment 1).

		**Conventional metaphor**						**Novel metaphor**					
**Answer**	**Predictors**	**Positive**		**Negative**		**Non-emotive**		**Positive**		**Negative**		**Non-emotive**	
**YES**		***β*** **=** **0.06**, ***R***^**2**^ **=** **0.64**, ***F***^**2**^ **=** **0.22**		***β*** **=** **0.66**, ***R***^**2**^ **=** **0.33**, ***F***^**2**^ **=** **0.37**		***β*** **=** **0.12**, ***R***^**2**^ **=** **0.65**, ***F***^**2**^ **=** **0.11**		***β*** **=** **0.09**, ***R***^**2**^ **=** **0.05**, ***F***^**2**^ **=** **0.28**		***β*** **=** **0.24**, ***R***^**2**^ **=** **0.47**, ***F***^**2**^ **=** **0.32**		***β*** **=** **0.14**, ***R***^**2**^ **=** **0.55**, ***F***^**2**^ **=** **0.34**	
		***β***	***t***	***β***	***t***	***β***	***t***	***β***	***t***	***β***	***t***	***β***	***t***
	Understandability	0.51	2.17^**^	0.22	3.43^**^	0.33	3.21^*^	0.41	1.09	0.44	3.14^**^	0.33	3.15^*^
	Convincingness	0.32	2.11^*^	0.27	1.79^*^	0.08	1.16	0.22	2.11^**^	0.35	2.97^**^	0.11	2.62
	Emotional appeal	0.22	2.27^**^	0.34	2.35^**^	0.11	1.72	0.44	2.67^*^	0.57	3.14^***^	0.43	2.19^**^
	Logical relation	0.41	2.14^**^	0.33	2.19^**^	0.19	1.53	0.32	2.11	0.46	2.54^**^	0.44	2.22^*^
	Ambiguity	0.17	2.7	0.13	2.66	0.21	2.72	0.08	2.17	0.52	2.19^**^	0.17	1.76
	Belief in the conclusion	0.21	2.31	0.45	2.81^***^	0.12	1.65	0.04	2.12	0.71	3.44^***^	0.61	2.46^**^
	Real world experience	0.32	2.19^*^	0.32	2.72	0.07	2.61	0.06	2.97	0.12	1.93	0.15	2.71
**NO**		***β*** ** = −0.02**, ***R***^**2**^ **=** **0.14**, ***F***^**2**^ **=** **0.18**		***β*** **=** **0.22**, ***R***^**2**^ **=** **0.31**, ***F***^**2**^ **=** **0.22**		***β*** **=** **0.07**, ***R***^**2**^ **=** **0.27**, ***F***^**2**^ **=** **0.21**		***β*** **=** **0.26**, ***R***^**2**^ **=** **0.27**, ***F***^**2**^ **=** **0.21**		***β*** ** = −0.02**, ***R***^**2**^ **=** **0.16**, ***F***^**2**^ **=** **0.12**		***β*** **=** **0.17**, ***R***^**2**^ **=** **0.07**, ***F***^**2**^ **=** **0.11**	
		***β***	***t***	***β***	***t***	***β***	***t***	***β***	***t***	***β***	***t***	***β***	***t***
	Understandability	0.19	2.1	0.56	3.41^**^	0.56	3.17^***^	0.32	3.16^**^	0.42	1.96^**^	0.51	2.45^**^
	Convincingness	0.11	2.71	0.43	2.79^*^	0.06	1.19	0.11	1.8	0.36	2.47^*^	0.10	2.71
	Emotional appeal	0.36	3.14^**^	0.21	1.16	0.15	3.32	0.25	2.19^*^	0.10	2.96	0.32	2.27^*^
	Logical relation	0.17	2.76	0.11	1.88	0.66	3.41^***^	0.22	1.14	0.22	3.27	0.46	3.46^*^
	Ambiguity	0.11	1.16	0.34	3.92^*^	0.12	3.11	0.27	2.89^*^	0.40	2.14^*^	0.50	2.17^**^
	Belief in the conclusion	0.09	3.5^*^	0.20	2.18	0.14	2.12	0.09	1.18	0.13	2.96	0.10	2.24
	Real world experience	0.05	2.18	0.21	1.67	0.38	3.44^*^	0.11	2.92	0.06	2.74	0.39	3.47^*^

* *p < 0.05*,

** *p < 0.01*,

*** *p < 0.001*.

A multiple linear regression was calculated to predict “yes” and “no” based on understandability, convincingness, emotional appeal, logical relation, ambiguity, belief in the conclusion and real world experience for novel and conventional metaphors. When “yes” was predicted it was found that seven predictors in positive (β = 0.06, *p* < 0.05), negative (β = 0.66, *p* < 0.05), and non-emotive (β = 0.12, *p* < 0.05) metaphorical middle term conditions were significant predictors in the case of conventional metaphors. The overall model fit for positive metaphor was *R*^2^ = 0.64, for negative metaphor was *R*^2^ = 0.33 and non-emotive metaphors was *R*^2^ = 0.65. When “yes” was predicted it was found that six predictors in positive (β = 0.09, *p* < 0.05), negative (β = 0.24, *p* < 0.05), and non-emotive (β = 0.14, *p* < 0.05) metaphorical middle term conditions were significant predictors in the case of novel metaphors. The overall model fit for positive metaphor was *R*^2^ = 0.05, for negative metaphor was *R*^2^ = 0.47 and non-emotive metaphor was *R*^2^ = 0.55. When “no” was predicted it was found that seven predictors in positive (β = −0.02, *p* < 0.05), negative (β = 0.22, *p* < 0.05), and non-emotive (β = 0.07, *p* < 0.05) metaphorical middle term conditions were significant predictors in the case of conventional metaphors. The overall model fit for positive metaphor was *R*^2^ = 0.14, for negative metaphor was *R*^2^ = 0.31 and non-emotive metaphors was *R*^2^ = 0.27. When “no” was predicted it was found that six predictors in positive (β = 0.26, *p* < 0.05), negative (β = −0.02, *p* < 0.05), and non-emotive (β = 0.17, *p* < 0.05) metaphorical middle term conditions were significant predictors in the case of novel metaphors. The overall model fit for positive metaphor was *R*^2^ = 0.27, for negative metaphor was *R*^2^ = 0.16 and non-emotive metaphor was *R*^2^ = 0.07.

The results showed that understandability was a significant predictor for both accepting and discarding the conclusion of the argument as following from the premises with both conventional and novel metaphors. When the conclusion was perceived to be following from the premises, for any argument with emotive metaphors, both convincingness and emotional appeal were significant predictors for committing the equivocation fallacy [Convincingness: CM+ (*t*_(89)_ = 2.11; *p* < 0.05), CM− (*t*_(89)_ = 1.79; *p* < 0.05), NM+ (*t*_(89)_ = 2.11; *p* < 0.01), and NM- (*t*_(89)_ = 2.97; *p* < 0.01); Emotional appeal: CM+ (*t*_(89)_ = 2.27; *p* < 0.01), CM− (*t*_(89)_ = 2.35; *p* < 0.01), NM+ (*t*_(89)_ = 2.67; *p* < 0.05), and NM- (*t*_(89)_ = 3.14; *p* < 0.001)]. Emotional appeal was a significant predictor also in the case of novel non-emotive metaphors (*t*_(89)_ = 2.19; *p* < 0.01). Interestingly, in the case of negatively valenced metaphors, also the perception of having found a logical relation in the argument and the belief in the conclusion, independent from the premises, were significant predictors for accepting the plausible conclusion [Logical relation: CM− (*t*_(89)_ = 2.19; *p* < 0.01), NM - (*t*_(89)_ = 2.54; *p* < 0.01); Belief in the conclusion: CM− (*t*_(89)_ = 2.81; *p* < 0.001), NM- (*t*_(89)_ = 3.44; *p* < 0.001)]. The perception of a logical relation was a significant predictor in the case of CM+ arguments (*t*_(89)_ = 2.14; *p* < 0.01), but not in the case of CM0 arguments. In the case of arguments with positively valenced metaphors, the belief in the conclusion was not a significant predictor for committing the equivocation fallacy, while it was significant in the case of NM0 arguments (*t*_(89)_ = 2.46; *p* < 0.001).

When the conclusion was seen not to be following from the premises, ambiguity was a significant predictor for all the arguments featuring novel metaphors [NM0 (*t*_(89)_ = 2.17; *p* < 0.01), NM+ (*t*_(89)_ = 2.89; *p* < 0.05), and NM- (*t*_(89)_ = 2.14; *p* < 0.05)], independent of their valence. In the case of negatively valenced metaphors, both ambiguity and convincingness were significant predictors for detecting the equivocation fallacy [Ambiguity: CM− (*t*_(89)_ = 3.92; *p* < 0.05), NM- (*t*_(89)_ = 2.14; *p* < 0.05); Convincingness: CM− (*t*_(89)_ = 2.79; *p* < 0.05), NM- (*t*_(89)_ = 2.47; *p* < 0.05)]. In the case of positively valenced metaphors, emotional appeal was a significant predictor for detecting the equivocation fallacy [Emotional appeal: CM+ (*t*_(89)_ = 3.14; *p* < 0.01), NM+ (*t*_(89)_ = 2.19; *p* < 0.05)]. In the case of non-emotive metaphors, having found a logical relation between premises and conclusion and real-world experience of similar arguments were significant predictors for detecting the fallacy [Logical relation: CM0 (*t*_(89)_ = 3.41; *p* < 0.001), NM0 (*t*_(89)_ = 3.46; *p* < 0.05); Real world experience: CM0 (*t*_(89)_ = 3.44; *p* < 0.05), NM0 (*t*_(89)_ = 3.47; *p* < 0.05)].

#### Discussion

The results of the Chi-squared test confirmed that participants mostly fail in the evaluation of syllogisms with metaphors and plausible conclusions (Ervas et al., 2018). The significant main effect of the metaphor type of the middle term on the evaluation of the arguments suggests that participants are more prone to commit the metaphoric fallacy when a conventional metaphor occurs in the first premise. This effect is independent of whether conventional metaphors are based on an emotive vehicle or not, and it can be explained by the metaphorical interference effect (MIE) (Glucksberg et al., 1982) in the truth evaluation of the first premise. When reading the premise with a conventional metaphor, we can assign it intuitive truth conditions, even though it is literally false. In a contextualist pragmatic perspective (Sperber and Wilson, 1986; Carston, 2002; Recanati, 2004, 2010), we understand the premise when we know the concrete circumstances of truth, which can depart from their literal ones. Processes of lexical modulation are supposed to adjust the literal meaning of the sentence and to provide an “adjusted” meaning, better fitting the context. This is also the case of conventional metaphors, whose vehicle encodes a source concept that is contextually modulated to generate an *ad hoc* concept in the proposition the speaker intends to communicate (Carston, 1997, 2002), corresponding to the intuitive truth-conditions she assigned. Diverging from the predictions of classical argumentation, we commit the metaphoric fallacy because we systematically and unawarely reject the “literal” truth conditions in the pragmatic process of conventional metaphor understanding, thus compromising the evaluation of the strength of the overall argument (Ervas et al., 2015).

Another route to metaphor understanding is followed when understanding novel metaphors (Carston, 2010, 2018). Especially in the case of unfamiliar and novel metaphors, the literal meaning of the vehicle would linger in the interpretation process of the premise, possibly requiring more contextual information to be intuitively perceived as true (Indurkhya, 2006, 2016; Carston, 2010; Ervas, 2019). However, the sentential context of the first premise, as well as the narrow syllogistic context of argumentation, would make novel metaphors' interpretation more easily recognized as false when compared to conventional metaphors. In a sense, novel (rather than conventional) metaphors are processed in a Gricean style in the first premise of the syllogism, as they are recognized from the very beginning as “patently false.” As there is a strict link between the truth conditions of the premises and the evaluation of the strength of the whole argument, it would be easier for participants to detect the metaphoric fallacy in the case of novel metaphors. Also, the suppression mechanism of the literal meaning should be more difficult in novel rather than in conventional metaphors interpretation (Gernsbacher and Faust, 1991; Gernsbacher et al., 2001; Rubio Fernandez, 2007). This would be crucial for the detection of a fallacy based on the equivocation between the metaphorical and the literal meaning of the middle term. Participants recognized ambiguity as a reason for rejecting the argument as unsound in all the cases of arguments with novel metaphors, independently of their emotive valence.

The significant main effect of the affective type of the middle term on the evaluation of the arguments and the *post-hoc* analysis' results suggest that participants are more prone to commit the metaphoric fallacy when a negatively valenced rather than a non-emotive metaphor occurs in the first premise. This effect is independent of whether the metaphors are conventional or novel and confirms previous studies on the effect of emotions on deductive reasoning: even though the validity of a conclusion should not depend on the emotional valence of the premises, it has been shown that negatively valenced contents are associated with decreased logicality compared to neutral contents (Lefford, 1946; Blanchette and Richards, 2004; Blanchette, 2006; Blanchette and Leese, 2011). Beyond confirming that the affective valence of the content influences normatively correct answers when reasoning about syllogisms, the results seem to confirm also the idea of a general negative effect of emotions on cognitive performance [see e.g., Lieberman et al. (2005)]. The answers provided by the participants to the seven questions - following the main question on the argument strength - let us better understand *why* participants were more prone to accept the metaphoric fallacy as sound in the case of emotive metaphors. When participants accepted the metaphoric fallacy as sound, its *emotional appeal* and *convincingness* had a crucial role, while the logical structure and real-life experience played a major role in the case of non-emotive metaphors, i.e., when participants did detect the metaphoric fallacy. However, emotional appeal is also a significant predictor for *detecting* the metaphoric fallacy in the case of positively valenced metaphor: this suggests that the positive valence of the metaphorical middle term might increase participants' logicality, thus questioning the idea that emotional content always undermines cognitive performance in reasoning tasks.

The absence of a significant interaction between the metaphor type and the affective type of the middle term suggests that the metaphorical framing and the affective framing might act independently. Nonetheless, when the affective values of the vehicle and the topic are coherent, both effects are in place in the evaluation of arguments featuring the metaphor. On the one hand, especially in the case of conventional metaphor, the metaphorical framing could have been covert (Thibodeau and Boroditsky, 2011, 2015) and thus more influential in guiding the participants to make evaluations consistent with the metaphorical heuristic rather than with logicality (Robins and Mayer, 2000). On the other hand, especially in the case of negatively valenced metaphors, the affective framing might have led participants to derail from the route to logicality, embracing heuristics when reasoning (Eliades et al., 2013). Previous research has argued in favor of the thesis that “emotions promote a form of reasoning which is less analytical and more heuristic-based” (Blanchette et al., 2018, p. 61), especially when the arguments are susceptible to the biasing effects of *conclusion believability* (Eliades et al., 2012). The results of the experiment confirm that, especially in the case of negatively valenced metaphors, participants' belief in the conclusion significantly leads them to accept the metaphoric fallacy as a sound argument, even thinking of having found a logical relation between premises and conclusion. However, controversial results on the influence of negatively valenced emotional content in reasoning can be found within the literature (Hofmann et al., 2009; MacKuen et al., 2010): it might be claimed that not necessarily the influence of negatively valenced stimuli leads participants to make more “errors.” Negatively valenced emotional content might have led the participants to find alternative reasons, regardless of their logical validity or of the argument strength, thus reinterpreting the premises of the arguments with metaphors to make sense of the believed conclusion. In this perspective, a global process of sense-making would have precedence over the analytic process in the evaluation of the “metaphoric fallacy” and lead the (re)interpretation of the overall argument as metaphorical.

### Experiment 2

The goal of the experiment was to test the evaluation of metaphoric fallacies, having a syllogistic form and - in their first premise - an emotive metaphor, whose vehicle has an emotional meaning *incoherent* with the emotional meaning of the topic. We aimed to understand whether the emotional meaning of the overall sentential metaphorical context of the first premise or the specific emotional meaning of the vehicle as middle term leads the participants to accept a fallacious argument as sound.

#### Participants

The participants (99 adults, 69 women, 33 men) were undergraduate students in Communication Science recruited at the University of Cagliari, had Italian as their first language, and normal/corrected vision. As in the first experiment, we checked for the participants' intuitive answers on different measures concerning the acceptability of argument conclusions, and excluded participants (*N* = 3) who had advanced training in logic and/or argumentation theory, resulting in 96 participants (63 women, 33 men, *M*_age_ = 23.90 years, *SD*_age_ = 8.39 years).

#### Materials

Participants were presented with a set of *N* = 36 arguments in Italian (Table A2 in Appendix), including the 12 literal arguments-fillers presented in the first experiment (Table A3 in Appendix). The arguments had the structure of syllogisms with plausible conclusions and contained *N* = 8 non-emotive metaphors, 4 CM0 and 4 NM0; *N* = 8 positively valenced metaphors, 4 CM+ and 4 NM+; *N* = 8 negatively valenced metaphors, 4 CM− and 4 NM-, in their first premise. Table 7 presents an example argument for each middle term condition.

**Table 7:** Examples of arguments in English for each middle term condition (Experiment 2).

	**Non-emotive metaphors (0)**	**Positively valenced metaphors (+)**	**Negatively valenced metaphors (-)**
CM	[P1] That girl is a *gem*. (*gemma*) [P2] A *gem* is a precious stone. [C] That girl is a precious stone.	[P1] A separation is a *thaw*. (*schiarita*) [P2] A thaw is the defreezing of ice. [C] A separation is the defreezing of ice.	[P1] Bravery is a *scar*. (*sfregio*) [P2] A *scar* is a wound. [C] Bravery is a wound.
NM	[P1] The rooster is a *pharaoh*. (*faraone*) [P2] A *pharaoh* is a leader. [C] The rooster is a leader.	[P1] Insolence is a *smile*. (*risata*) [P2] A *smile* is an expression of joy. [C] Insolence is an expression of joy.	[P1] Religion is a *scald*. (*ustione*) [P2] A *scald* is a serious burn. [C] Religion is a serious burn.

The set of syllogisms with non-emotive metaphors was the same as used in the first study, and the set of syllogisms with emotive metaphors had the same second premises of the syllogisms as in the first study. The emotive metaphors had the same metaphor vehicles as middle terms of the syllogisms, but different metaphor topics when compared to the emotive metaphors used in Experiment 1. In Experiment 2, the emotive metaphors in the first premises of the syllogisms were emotionally incoherent. Consequently, the conclusions differed from the conclusions of the syllogisms in Experiment 1, because they connect different metaphor topics to the last term of each second premise. Still, the evaluation of the syllogisms depends on the same middle terms, which are the metaphor vehicles.

#### Procedure

We collected the data through an online form, where all the participants signed their informed consent and provided information about their gender, age, language, and education. We followed the same procedure used in Experiment 1, asking participants to read the instructions, complete the practice block and to evaluate the randomly presented arguments. For each argument, the participants were asked to evaluate whether the conclusion of the argument followed from the premises (“Yes” or “No” answers) and to rate the arguments on a 1–5 Likert scale (1 = least likely, 5 = most likely), based on the answers to the measures: (1) Understandability; (2) Convincingness; (3) Emotional appeal; (4) Logical relation; (5) Ambiguity; (6) Belief in the conclusion; (7) Real-world experience. Participants employed ca. 30 min to complete the experiment.

#### Results

All the data collected are available at the following **OSF address** (https://osf.io/jzpva/?view_only=8edc3b523cbb4afba4bd71978d847a48). Mean and standard deviation for accuracy are presented in Table 8. We first checked whether the participants correctly answered the fillers, i.e., the literal arguments, to understand whether they actually have a basic ability in detecting clearly strong vs. weak arguments. As in the first experiment, the participants performed almost at ceiling in evaluating literal arguments. The Chi-squared test suggested that they provided significantly more correct than incorrect answers to literal arguments (*p* < 0.001; LS: χ^2^ = 140.29, φ = 0.85; LW: χ^2^ = 95.41, φ = 0.70; LP: χ^2^ = 62.00, ϕ=0.57). We performed paired *t*-tests between literal arguments with plausible conclusions and clearly strong vs. weak literal arguments: the results suggested that fallacious arguments with plausible conclusions were more difficult to detect when compared to both clearly strong (*p* < 0.001) and clearly weak arguments (*p* < 0.01). The Chi-squared test also suggested that participants provided more incorrect than correct answers to arguments with metaphors (*p* < 0.001; CM0: χ^2^ = 81.91, φ = 0.65; CM+: χ^2^ = 52.76, φ = 0.52; CM−: χ^2^ = 40.48, φ = 0.46; NM0: χ^2^ = 44.50, φ = 0.48; NM+: χ^2^ = 47.35, φ = 0.50; NM-: χ^2^ = 67.50, φ = 0.59).

**Table 8:** Mean (M) and standard deviation (SD) values of correct answers for each middle term condition (Experiment 2).

	**Positively valenced**		**Negatively valenced**		**Non-emotive**	
	***M***	***SD***	***M***	***SD***	***M***	***SD***
CM	0.23	0.30	0.30	0.34	0.15	0.26
NM	0.28	0.32	0.17	0.23	0.24	0.27
**Literal**	**Strong arguments**		**Weak arguments**		**Arguments with plausible conclusion**	
	0.97	0.10	0.89	0.21	0.80	0.24

A two-way ANOVA was performed for accuracy, to evaluate the main effects of the metaphorical and affective middle term type and the effect of their interaction on the evaluation of the fallacious arguments. The results of the main effects are reported in Table 3, comparing them to the results of the first experiment. Differently from the first experiment, the ANOVA results showed no significant main effects of the metaphorical [*F*_(1,94)_ = 0.01; *p* = 0.91; η^2^ = 0.00] and affective [*F*_(2,93)_ = 2.11; *p* = 0.12; η^2^ = 0.007] middle term type, but instead a significant interaction effect between them [*F*_(2,91)_ = 7.89; *p* < 0.001; η^2^ = 0.027]. A Tukey's test, corrected for multiple comparisons, was performed to assess the statistical significance of the interaction effect (see Table 9 for all the results). The *post-hoc* analysis revealed a higher level of accuracy especially in the evaluation of CM− arguments, when compared to both CM0 arguments [*t*_(94)_ = 0.60; *p* = 0.004] and NM- arguments [*t*_(94)_ = 0.52; *p* = 0.02]. Furthermore, it showed a significant difference between the level of accuracy in the evaluation of NM+ arguments when compared to CM0 arguments, which received less correct answers [*t*_(94)_ = 0.51; *p* = 0.02].

**Table 9:** *t/p*-values for accuracy, comparing middle term conditions (Experiment 2).

	**Comparisons**	***t***	***p***
C(Metaphorical): C (Affective)	CM0/CM- middle terms	−0.60	0.004
	CM+/CM- middle terms	−0.29	0.49
	NM-/CM- middle terms	−0.52	0.02
	NM0/CM- middle terms	−0.25	0.66
	NM+/CM- middle terms	−0.093	0.99
	CM+/CM0 middle terms	0.31	0.41
	NM-/CM0 middle terms	0.08	0.99
	NM0/CM0 middle terms	0.35	0.27
	NM+/CM0 middle terms	0.51	0.02
	NM-/CM+ middle terms	−0.22	0.73
	NM0/CM+ middle terms	0.04	0.99
	NM+/CM+ middle terms	0.19	0.84
	NM0/NM- middle terms	0.27	0.57
	NM+/NM- middle terms	0.42	0.10
	NM+/NM0 middle terms	0.15	0.93

*Positively valenced (CM+), negatively valenced (CM-), non-emotive (CM0) conventional metaphor. Positively valenced (NM+), negatively valenced (NM-), non-emotive (NM0) novel metaphor*.

A multiple linear regression was calculated to predict “yes” and “no” based on understandability, convincingness, emotional appeal, logical relation, ambiguity, belief in the conclusion and real world experience for novel and conventional metaphors (see M and SD values for each measure in Table 10). Table 11 presents the results of the linear regression for all middle term conditions. When “yes” was predicted it was found that four predictors in positive (β = 0.16, *p* < 0.05), negative (β = 0.44, *p* < 0.05), and non-emotive (β = 0.37, *p* < 0.05) metaphorical middle term conditions were significant predictors in the case of conventional metaphors. The overall model fit for positive metaphor was *R*^2^ = 0.24, for negative metaphor was *R*^2^ = 0.21 and non-emotive metaphors was *R*^2^ = 0.25. When “yes” was predicted it was found that seven predictors in positive (β = 0.11, *p* < 0.05), negative (β = 0.27, *p* < 0.05), and non-emotive (β = 0.27, *p* < 0.05) metaphorical middle term conditions were significant predictors in the case of novel metaphors. The overall model fit for positive metaphor was *R*^2^ = 0.07, for negative metaphor was *R*^2^ = 0.24 and non-emotive metaphors was *R*^2^ = 0.35. When “no” was predicted it was found that four predictors in positive (β = 0.19, *p* < 0.001), negative (β = 0.12, *p* < 0.01), and non-emotive (β = 0.13, *p* < 0.05) metaphorical middle term conditions were significant predictors in the case of conventional metaphors. The overall model fit for positive metaphor was *R*^2^ = 0.04, for negative metaphor was *R*^2^ = 0.41 and non-emotive metaphors was *R*^2^ = 0.17. When “no” was predicted it was found that seven predictors in positive (β = 0.16, *p* < 0.05), negative (β = −0.07, *p* < 0.05), and non-emotive (β = 0.07, *p* < 0.05) metaphorical middle term conditions were significant predictors in the case of novel metaphors. The overall model fit for positive metaphor was *R*^2^ = 0.13, for negative metaphor was *R*^2^ = 0.11 and non-emotive metaphor was *R*^2^ = 0.19.

**Table 10:** Mean (M) and standard deviation (SD) values of each predictor for each middle term condition (Experiment 2).

**Predictor**	**CM+**		**CM-**		**CM0**		**NM+**		**NM-**		**NM0**	
	***M***	***SD***	***M***	***SD***	***M***	***SD***	***M***	***SD***	***M***	***SD***	***M***	***SD***
Understandability	2.91	0.77	2.52	0.78	3.48	0.76	2.78	0.74	3.08	0.78	2.77	0.81
Convincingness	2.42	0.64	2.04	0.68	3.14	0.72	2.25	0.61	2.76	0.62	2.27	0.76
Emotional appeal	1.92	0.75	1.51	0.57	2.29	0.79	1.95	0.72	1.85	0.70	1.49	0.47
Logical relation	2.86	0.70	2.53	0.76	3.23	0.72	2.61	0.69	2.98	0.71	2.69	0.74
Ambiguity	2.35	0.62	2.60	0.71	2.13	0.70	2.40	0.72	2.20	0.61	2.51	0.76
Belief in the conclusion	2.41	0.70	1.93	0.64	3.23	0.73	2.20	0.68	2.64	0.71	2.51	0.77
Real world experience	2.33	0.89	1.90	0.80	3.04	0.84	2.19	0.79	2.58	0.88	2.07	0.87

*Positively valenced (CM+), negatively valenced (CM-), non-emotive (CM0) conventional metaphor. Positively valenced (NM+), negatively valenced (NM-), non-emotive (NM0) novel metaphor*.

**Table 11:** *t/p*-values for each predictor in the evaluation of arguments, comparing the middle term conditions (Experiment 2).

		**Conventional metaphor**						**Novel metaphor**					
**Answer**	**Predictors**	**Positive**		**Negative**		**Non-emotive**		**Positive**		**Negative**		**Non-emotive**	
**YES**		*****β***** **=** **0.16**, ***R***^****2****^ **=** **0.24**, ***F***^****2****^ **=** **0.10**		*****β***** **=** **0.44**, ***R***^****2****^ **=** **0.21**, ***F***^****2****^ **=** **0.33**		*****β***** **=** **0.37**, ***R***^****2****^ **=** **0.25**, ***F***^****2****^ **=** **0.28**		*****β***** **=** **0.11**, ***R***^****2****^ **=** **0.07**, ***F***^****2****^ **=** **0.23**		*****β***** **=** **0.27**, ***R***^****2****^ **=** **0.24**, ***F***^****2****^ **=** **0.11**		*****β***** **=** **0.27**, ***R***^****2****^ **=** **0.35**, ***F***^****2****^ **=** **0.33**	
		**β**	***t***	**β**	***t***	**β**	***t***	**β**	***t***	**β**	***t***	**β**	***t***
	Understandability	0.33	2.91^*^	0.34	2.29^**^	0.29	2.51^*^	0.55	3.15^***^	0.47	2.66^**^	0.57	2.97^*^
	Convincingness	0.29	3.2^**^	0.22	3.4^*^	0.33	3.71^*^	0.43	2.72^**^	0.55	3.61^*^	0.30	3.1
	Emotional appeal	0.22	2.7^*^	0.09	1.96^*^	0.21	2.66	0.20	2.37^*^	0.48	2.01^**^	0.08	1.6
	Logical relation	0.20	1.47	0.21	1.15	0.07	2.42	0.11	1.18	0.44	3.77^*^	0.31	3.79^**^
	Ambiguity	0.32	2.51	0.17	2.9	0.12	2.01	0.09	2.91	0.53	1.9^**^	0.04	1.46
	Belief in the conclusion	0.17	2.33	0.67	1.91^***^	0.11	3.07	0.07	2.12	0.40	2.7^*^	0.05	2.1
	Real world experience	0.11	1.56	0.22	1.41	0.20	2.4	0.13	2.11	0.21	1.9	0.24	2.7^*^
NO		***β*** **=** **0.19**, ***R***^**2**^ **=** **0.04**, ***F***^**2**^ **=** **0.34**		***β*** **=** **0.12**, ***R***^**2**^ **=** **0.41**, ***F***^**2**^ **=** **0.21**		***β*** **=** **0.13**, ***R***^**2**^ **=** **0.17**, ***F***^**2**^ **=** **0.11**		***β*** **=** **0.16**, ***R***^**2**^ **=** **0.13**, ***F***^**2**^ **=** **0.12**		***β*** ** = −0.07**, ***R***^**2**^ **=** **0.11**, ***F***^**2**^ **=** **0.17**		***β*** **=** **0.07**, ***R***^**2**^ **=** **0.19**, ***F***^**2**^ **=** **0.21**	
		***β***	***t***	***β***	***t***	***β***	***T***	***β***	***t***	***β***	***t***	***β***	***t***
	Understandability	0.24	1.4	0.36	2.74^**^	0.33	3.47^***^	0.76	3.04^**^	0.30	2.01^**^	0.41	2.97^**^
	Convincingness	0.09	2.72	0.29	2.4^**^	0.14	2.07	0.10	1.14	0.21	2.71^*^	0.11	3.41
	Emotional appeal	0.19	2.47	0.31	2.8	0.19	2.11	0.36	2.9^*^	0.06	1.13	0.15	3.07
	Logical relation	0.57	2.22^***^	0.09	1.61	0.41	2.41^**^	0.09	1.8	0.03	1.71	0.28	2.4^**^
	Ambiguity	0.22	1.91	0.11	2.11	0.22	3.53	0.28	2.17^*^	0.17	1.72	0.09	2.02
	Belief in the conclusion	0.19	1.71	0.27	2.34	0.05	1.17	0.07	2.44	0.29	2.7^*^	0.30	1.26
	Real world experience	0.07	1.01	0.07	2.02	0.37	1.21^*^	0.10	2.21	0.47	3.4	0.28	2.48^*^

* *p < 0.05*,

** *p < 0.01*,

*** *p < 0.001*.

As in the first experiment, the results of the linear regression showed that understandability was a significant predictor for both accepting and discarding the conclusion of the argument as following from the premises in both conventional and novel metaphors. When the conclusion was seen to be following from the premises, for any argument with conventional emotive metaphor, both convincingness and emotional appeal were significant predictors for committing the equivocation fallacy [Convincingness: CM+ (*t*_(94)_ = 3.2; *p* < 0.01), CM− (*t*_(94)_ = 3.4; *p* < 0.05); Emotional appeal: CM+ (*t*_(94)_ = 2.7; *p* < 0.05), CM− (*t*_(94)_ = 1.96; *p* < 0.05)]. Convincingness was also a significant predictor for committing the fallacy in the case of CM0 arguments (*t*_(94)_ = 3.71; *p* < 0.05). Emotional appeal was also a significant predictor for accepting the fallacious argument in the case of NM- arguments (*t*_(94)_ = 2.01; *p* < 0.01), as well as the perception of having found a logical relation between premises and conclusion (*t*_(94)_ = 3.77; *p* < 0.05) and some ambiguity in the argument (*t*_(94)_ = 1.9; *p* < 0.01). In the case of NM0 arguments, having found a logical relation between premises and conclusion (*t*_(94)_ = 3.79; *p* < 0.01) and real-world experience of similar arguments (*t*_(94)_ = 2.7; *p* < 0.05) were significant predictors for committing the fallacy. Interestingly, the belief in the conclusion was a significant predictor in the case of arguments with negatively valenced metaphors [CM− (*t*_(94)_ = 1.91; *p* < 0.001), NM- (*t*_(94)_ = 2.7; *p* < 0.05)].

When the conclusion was detected as not following from the premises, convincingness was a significant predictor in the case of arguments with negatively valenced metaphors [CM− (*t*_(94)_ = 2.4; *p* < 0.01), NM- (*t*_(94)_ = 2.71; *p* < 0.05)]. Logical relation was significant predictor for detecting the metaphoric fallacy in the case of CM+ (*t*_(94)_ = 2.22; *p* < 0.001), CM0 (*t*_(94)_ = 2.41; *p* < 0.01) and NM0 (*t*_(94)_ = 2.4; *p* < 0.01) arguments. Ambiguity and emotional appeal were instead significant predictor for detecting the metaphoric fallacy in the case of positively valenced novel metaphors [Ambiguity: *t*_(94)_ = 2.17; *p* < 0.05; Emotional appeal: *t*_(94)_ = 2.9; *p* < 0.05]. Real world experience of similar arguments was a significant predictor in the case of arguments with non-emotive metaphors [CM0: *t*_(94)_ = 1.21; *p* < 0.05, NM0: *t*_(94)_ = 2.48; *p* < 0.05] and negatively valenced novel metaphors (*t*_(94)_ = 3.4; *p* < 0.01).

#### Discussion

The results confirm that participants mostly fail in the evaluation of the metaphoric fallacy. However, they suggest that the affective valence of the overall metaphor, rather than the vehicle alone as middle term, influences the detection of the metaphoric fallacy. When the first premise is affectively incoherent and more difficult to make sense of, neither the metaphorical nor the affective framing significantly influence participants' evaluation. This suggests that the initial incoherence of the arguments make participants abandon the global heuristic process at work in both metaphorical and affective framing in arguments' assessment. As suggested in argumentation theory by Walton (1996), the metaphoric fallacy is a special kind of equivocation fallacy where a global process of metaphorical interpretation is in place, differing from the mere process of disambiguation required by literal middle terms in the detection of the equivocation fallacy. A global interpretation would involve an evaluation of the overall first premise featuring the metaphor, including both the topic and the vehicle. Global aspects prevailed when participants committed the fallacy: the emotional appeal and the convincingness of the argument in the case of emotive metaphors and, as in the first experiment, the belief in the conclusion when assessing syllogisms with negatively valenced metaphors.

When it is more difficult to make sense of the *affective incoherence* of the metaphor in the first premise, the metaphorical and the affective framing interact, *increasing participants' logicality* not only in the case of novel positively valenced metaphors (compared to conventional non-emotive metaphors), but also in the case of conventional negatively valenced metaphors (compared to conventional non-emotive and novel negatively valenced metaphors). More correct answers are provided in those conditions, suggesting a more nuanced picture, where emotive metaphors do not have the usual deleterious role in reasoning. In the case of NM+ arguments, participants detected the ambiguity of the fallacy, and in the case of CM−, participants checked for the convincingness of the argument itself, without being affected by the believability of the conclusion (as in the case of NM- arguments). This suggests that the initial incoherence of the arguments makes participants more vigilant in specific and local cases where the type of metaphor and its affective valence did play a role in avoiding the negative consequences associated with endorsing unsound arguments. Thus, a more analytical style of reasoning is embraced by the participants: in the case of novel positively valenced metaphors, the evident distance between the literal and the metaphorical meaning is more easily detected; in the case of conventional negatively valenced metaphors, the intention to convince the participants make them more doubting and less likely to endorse the believable but unsound conclusion. This more systematic information processing style probably required the allocation of extra attention and more cognitive resources (Forgas, 1995), requiring to assess both the metaphorical and affective effects.

### General Discussion

Both metaphors and emotions have been said to entail a negative effect on reasoning. The studies highlight how the picture is more complicated: not always the metaphorical framing interacts with the affective framing leading to a decreased logicality and not always a decreased logicality is justified by a deleterious use of the heuristics entailed by the metaphorical and the affective framing in reasoning. This could be explained by dual-processing models [see e.g., Kahneman (2003), Evans (2008), and Evans and Frankish (2009)], envisaging two distinct inferential (automatic vs. controlled) processes: notoriously, System 1 and System 2. The first includes quick associative, emotional and heuristic processes working in a parallel, effortless and unconscious way; the second includes slow rule-governed, neutral and content-blind processes working in a serial, effortful and often conscious way (Ervas et al., 2015). Metaphors can be seen as heuristic, System 1-type processes that never guarantee the preservation of truth in deductive reasoning tasks up to System 2 (Fischer, 2014; Keefer and Landau, 2016), though intuitively leading to genuinely new knowledge *via* a creative argumentation style (Schn, 1993; Leung et al., 2012). In this perspective, as pointed out by Haack (1994, p. 4), metaphors can be seen as “cognitively vital” and illuminating, but “can also be feeble; can be exploited to the purpose of persuading by emotional appeal rather than rational argument.” As argued in recent psychological literature (Citron and Goldberg, 2014, p. 9), metaphors are “more emotionally engaging than literal expressions,” especially when they are grounded in perceptual and sensorimotor representations (Indurkhya, 1992, 1994; Gibbs, 2006), recalling people's experiential knowledge rather than more abstract and analytical constructs. In the same vein, emotions have been said to elicit System 1-type processes, depleting available cognitive resources at the expense of System 2, thus affecting analytical reasoning and decreasing logicality to a greater extent (Channon and Baker, 1994; Kensinger and Corkin, 2003; De Neys, 2012).

However, the relationship between System 1 and System 2 processes is largely understood in terms of competition or conflict:embodiment plays a role only in heuristics and/or emotional processes, while “proper” reasoning processes still deserve a superior function of control and/or revision. From a philosophical point of view, dual-processing models have been criticized as proposing an anachronistic view of mind, having a disembodied normative System 2 working in opposition to an embodied System 1 (Marraffa, 2014). More recent “integrated” dual-processing models offer an alternative view of reasoning, based on the coordination rather than competition between different inferential processes [see e.g., Moshman (2004), Mercier and Sperber (2011), Carruthers (2011), Fletcher and Carruthers (2012), Baumard and Boyer (2013), and Rossi (2014)]. From a psychological point of view, it has been pointed out that emotional heuristic processes are not always in contrast with normatively-correct or analytical reasoning processes. A number of studies showed that positive emotional content improves performance on conditional and/or syllogistic reasoning (Isen et al., 1987; Melton, 1995). Especially when this content is *relevant* to the participants' prior real-life experience and/or their current emotional state (Blanchette, 2006; Johnson-Laird et al., 2006; Blanchette et al., 2007; Blanchette and Campbell, 2012; Caparos and Blanchette, 2016), there is a reduction of the classic belief bias effect on arguments' evaluation in the case of negative emotional content (Goel and Vartanian, 2011). Rather than contraposing emotional and reasoning processes, Blanchette et al. concluded that, depending on the *emotional relevance* to the evaluator, the same emotional content might have either an “incidental affect” (non-relevant) or a more “integral affect” (relevant) on the reasoning task, respectively enhancing the heuristic processes of System 1 and the analytical processes of System 2 [Blanchette and Richards, 2010; Huntsinger, 2013; Caparos and Blanchette, 2015; but see Jung et al. (2014) for different results].

The present study shows that another element, *affective coherence*, needs to be considered to have a more comprehensive (but also more nuanced) view of the interaction between emotional content and metaphorical (heuristic) processes and rule-governed (normative) processes. The comparison between the main results of the first and the second experiments (Table 3) suggests that affective coherence is crucial to understand the main effects of the metaphor type and the affective type of the middle term on the evaluation of the arguments, and their interaction. When the first premises are *affectively coherent* both the metaphorical and the affective framing significantly influence the argument evaluation. However, when the first premises are *affectively incoherent*, neither the metaphorical nor the affective framing significantly influence the argument evaluation. The results suggest that the *affective coherence* of the vehicle and the topic is responsible for the emergence of both the framing effects in the syllogisms' evaluation. Different from relevance, affective coherence concerns the emotional content of the metaphor's vehicle/topic, rather than the relationship with the evaluators' emotional state. The results suggest that affective coherence is more basic and primitive than relevance, determining the range of possible metaphorical and affective framing effects and influencing the overall participants' attempt to make sense of the argument. Affective coherence has an impact on the early stages of semantic sentence processing, automatically influencing sense-making (Schauenburg et al., 2019). Other studies showed that affective coherence also leads to better recall of the content (Richards and Gross, 1999), serving “as evidence of the appropriateness of affective concepts that come to mind” (Centerbar et al., 2008, p. 560). The condition of affective coherence induces “affective certainty” (Tamir et al., 2002), which “allow participants to devote themselves fully to the task at hand” (Clore and Schnall, 2008, p. 3).

The results of the experiments also suggest that, for reasoning with emotive metaphors, affective coherence promotes more holistic, *global and heuristic processes* when both metaphorical framing and affective framing contribute to creatively make sense of the overall argument, diverging to strict normative rules of logic in the metaphoric fallacy evaluation. However, affective incoherence makes global processes of sense-making more difficult to be carried out, neutralizing both general metaphorical and affective framing effects and improving logicality in *local and analytical processes*, where the framing effects interact to get rid of ambiguity or the believability of the conclusion, usually deviating normative reasoning. The direct emotional impact comes first (Zajonc, 1980) and determines the attitudes toward what is coming next (Petty and Cacioppo, 1986b; Petty and Briñol, 2015) and the reasoning style of the evaluator: affective coherent representations make participants more certain and prone to read/listen to what may be coming next, while affective incoherent representations make participants more dubious and vigilant toward possible rule violations. Affective incoherence vs. coherence draw us to play two different games in reasoning with emotive metaphors, which could be called respectively the “doubting game” and the “believing game” (Elbow, 1998). The doubting game questions previous beliefs and preconceptions, especially when self-evident, as well as “systems of commonplaces” associated with metaphors, relying only on literal truth and necessary consequences. The believing game accepts believable conclusions to figure out new or revitalized meanings in the premises and make sense of the whole argument metaphorically (Elbow, 1998). By inhibiting the doubting game operating in rule-governed deduction, the believing game might lead to knowing something new or to see some previously unveiled alternatives. The games are not exclusive even though they cannot be played simultaneously, as some affective situations may be more appropriate than others to play the believing vs. the doubting game.

Depending on the affective coherence vs. incoherence between the concepts involved in metaphor understanding, the interplay between metaphorical and affective framing could be consistent with both the possibility that emotional stimuli activated concepts or images that required additional processing resources in favor of the heuristic processes of the System 1 (at the expenses of the System 2), and the possibility that the concepts activated by emotional stimuli overlap with the representations necessary for the resolution of the reasoning problem, thus favoring System 2 processes. Specifically, we can now answer the questions leading us till here:

Q1. Under what conditions does the double framing effect of emotive metaphors mostly influence the evaluation of the argument, leading to a fallacy of equivocation?
R1. In the condition of affective incoherence rather than affective coherence, the metaphorical and the affective framing mostly interact, influencing the evaluation of the argument, and increasing the ability to identify the fallacy of equivocation. Under affective uncertainty, participants are more prone to questioning the premises of the arguments and the believability of the conclusion, improving System 2-type analytical and local processes at the expense of System 1-type global processes. The double framing effect of emotive metaphors is most significant in reasoning with conventional negatively valenced metaphors and novel positively valenced metaphors, though for different reasons. For conventional negatively valenced metaphors, the arguments' level of convincingness led participants to discard them as fallacious. For positively valenced metaphors, arguments' emotional appeal and the ambiguity between the metaphorical and literal meanings of the premises led participants to identify the equivocation fallacy.
Q2. Under what conditions does the metaphorical framing influence the evaluation of an argument? Are participants more prone to commit a fallacy of equivocation in the case of conventional metaphors or in the case of novel metaphors?
R2. In the condition of affective coherence rather than affective incoherence, the metaphorical framing influences the overall evaluation of an argument, leading to making sense of the premises in light of the believed conclusion *via* a global, System 1-type heuristic process. Especially in the case of conventional metaphors, which are not consciously processed as metaphors and whose intuitive truth conditions are more difficult to question, participants are more prone to disattend System 2-type reasoning, thus falling into the equivocation fallacy.
Q3. Under what conditions does the affective framing influence the evaluation of the argument? Are participants more prone to commit a fallacy of equivocation in the case of negatively valenced metaphors or in the case of positively valenced metaphors?
R3. In the condition of affective coherence rather than affective incoherence, the affective framing influences the overall evaluation of an argument *via* System 1-type processes, i.e., leveraging on the convincingness and emotional appeal of the arguments featuring emotive metaphors. Especially in the case of negatively valenced metaphors, participants are more prone to committing the equivocation fallacy, accepting the believable conclusion and finding a premise/conclusion connection alternative to the corrected System 2-type logical connection.

## Conclusions

Far from just leading to fallacies of reasoning, both metaphors and emotions entail a framing effect that makes us see things under a new perspective, influencing our decisions and evaluations in many, and sometimes unexpected, ways. Rather than always contrasting normative reasoning, the double framing effect can promote creative heuristic processes and increase logicality. Emotive metaphors do entail a double framing effect in argumentation, coming from their being metaphors and having an emotive (evaluative) connotation, as hypothesized (H1), but they do not lead to equivocation fallacies as expected. Depending on the contextual affective coherence and incoherence of the first premise, respectively, the double framing effect led participants to globally interpret the argument as metaphoric, or locally detect the ambiguity which leads to the equivocation fallacy. The metaphorical framing effect is stronger for conventional metaphors and the affective framing effect for negatively valenced metaphors, as hypothesized (respectively in H2 and H3), but they are both significant only when there is a more basic affective coherence between the vehicle and the topic of the metaphor, which also makes it possible to make more sense of the argument as a whole.

The experiments were limited in only considering coherence with respect to the affective valence of metaphors. Further research should consider also the affective coherence with respect to arousal and intensity (Clore and Schnall, 2005), which could provide information about other dimensions of the affective as well as metaphorical framing effect in reasoning with emotive metaphors. More ecologically valid studies could be designed to include the relevance of emotive metaphors to participants' emotional states and/or specific relevant properties of the metaphor that could be crucially extended in the second premises, thus further promoting a holistic interpretation of the metaphoric fallacy. Further research is also needed to understand whether, as Walton (1996) suggested, emotive metaphors are not as responsible for ambiguity fallacies as for other clarity fallacies. Finally, the double framing effect of emotive metaphors might be further investigated in fallacious arguments which explicitly appeal to emotions, to check whether conventional negative metaphors and novel positive metaphors still help to detect persuasive but fallacious arguments. This might also help us to better understand when people play the believing game or the doubting game (Elbow, 1998) in reasoning with emotive metaphors.

## Data Availability Statement

The datasets presented in this study can be found in online repositories. The names of the repository/repositories and accession number(s) can be found at: https://osf.io/jzpva/?view_only=8edc3b523cbb4afba4bd71978d847a48.

## Ethics Statement

The studies involving human participants were reviewed and approved by the ethics committee of the Department of Education, Psychology, Philosophy, University of Cagliari. The participants provided their written informed consent to participate in this study.

## Author Contributions

FE, MR, AO, and BI: design. FE: construction of the materials and manuscript writing. FE, MR, and AO: data collection. FE and AO: data analysis (respectively analysis of variance and regression analysis). All authors provided feedback on the draft and approved the final version of the manuscript.

## Conflict of Interest

The authors declare that the research was conducted in the absence of any commercial or financial relationships that could be construed as a potential conflict of interest.

## Appendix

**Table A1:** Mean (M) and standard deviation (SD) for each measure of the metaphors in Experiment 1 and Experiment 2.

	**Emotional meaning**		**Familiarity**		**Meaningfulness**		**Comprehension difficulty**	
	**M**	**SD**	**M**	**SD**	**M**	**SD**	**M**	**SD**
**Rating study (Experiment 1)** ***Metaphors***								
**CM+**	4.36	0.48	3.48	1.04	4.43	0.52	1.47	0.47
**NM+**	4.29	0.49	2.71	0.73	4.08	0.61	1.75	0.63
**CM-**	1.97	0.80	3.33	1.00	4.30	0.78	1.71	0.78
**NM-**	1.89	0.71	2.48	0.97	3.56	0.77	2.46	0.88
**CM0**	3.01	0.74	3.69	0.81	4.17	0.61	1.68	0.58
**NM0**	2.99	0.59	2.20	0.96	3.29	0.74	2.65	0.86
**Rating study (Experiment 2)** ***Metaphors***								
**CM+**	4.10	0.51	3.11	0.81	3.96	0.49	1.88	0.55
**NM+**	4.09	0.52	2.02	0.73	3.82	0.58	2.15	0.53
**CM-**	1.98	0.45	3.02	0.86	3.29	0.70	2.64	0.75
**NM-**	1.92	0.55	2.03	0.77	3.17	0.55	2.76	0.67
**CM0**	3.05	0.68	3.67	0.93	4.01	0.41	1.89	0.44
**NM0**	2.93	0.51	2.13	0.88	3.29	0.50	2.48	0.60

*Positively valenced (CM+), negatively valenced (CM-), non-emotive (CM0) conventional metaphor. Positively valenced (NM+), negatively valenced (NM-), non-emotive (NM0) novel metaphor*.

**Table A2:** Table of materials in Italian for each middle term condition in Experiment 1 and Experiment 2.

	**Non-emotive metaphors (0)**	**Positive-valenced metaphors (+)**	**Negative-valenced metaphors (–)**
		**Experiment 1**	
CM	[P1] Il corpo è un tempio.	[P1] Un caffè è un ristoro.	[P1] Un insulto è uno sfregio.
	[P2] Un tempio è sacro.	[P2] Un ristoro ridà benessere.	[P2] Uno sfregio è una ferita.
	[C] Il corpo è sacro.	[C] Un caffè ridà benessere.	[C] Un insulto è una ferita.
	[P1] Una risata è un farmaco.	[P1] Lo sport è la vitalità.	[P1] Una separazione è un trauma.
	[P2] Un farmaco serve alla salute.	[P2] La vitalità è energia.	[P2] Un trauma è un evento violento.
	[C] Una risata serve alla salute.	[C] Lo sport è energia.	[C] Una separazione è un evento violento.
	[P1] La vita è un capitolo.	[P1] La tolleranza è un antidoto.	[P1] Una sconfitta è un tonfo.
	[P2] Un capitolo è una parte di un libro.	[P2] Un antidoto contrasta i veleni.	[P2] Un tonfo è una caduta a terra.
	[C] La vita è una parte di un libro.	[C] La tolleranza contrasta i veleni.	[C] Una sconfitta è una caduta a terra.
	[P1] Quella ragazza è una gemma.	[P1] La pace è una schiarita.	[P1] Il traffico è un ingorgo.
	[P2] Una gemma è una pietra preziosa.	[P2] Una schiarita è il ritorno del sereno.	[P2] Un ingorgo è un intasamento.
	[C] Quella ragazza è una pietra preziosa.	[C] La pace è il ritorno del sereno.	[C] Il traffico è un intasamento.
NM	[P1] Il gallo è un faraone.	[P1] La libertà è una risata.	[P1] Lo smog è un accusato.
	[P2] Un faraone è un capo.	[P2] Una risata è un'espressione di gioia.	[P2] Un accusato è un possibile colpevole.
	[C] Il gallo è un capo.	[C] La libertà è un'espressione di gioia.	[C] Lo smog è un possibile colpevole.
	[P1] Un grido è un megafono.	[P1] Il tempo è un alleato.	[P1] Il circo è una slealtà.
	[P2] Un megafono emette suoni.	[P2] Un alleato è un amico.	[P2] Una slealtà è un'attività disonesta.
	[C] Un grido emette suoni.	[C] Il tempo è un amico.	[C] Il circo è un'attività disonesta.
	[P1] Il cuore è un'anfora.	[P1] La serenità è un vaccino.	[P1] La politica è un tugurio.
	[P2] Un'anfora trasporta liquidi.	[P2] Un vaccino rende immuni.	[P2] Un tugurio è in pessime condizioni.
	[C] Il cuore trasporta liquidi.	[C] La serenità rende immuni.	[C] La politica è in pessime condizioni.
	[P1] Il lavoro è un badile.	[P1] La felicità è un brillio.	[P1] Un tradimento è un'ustione.
	[P2] Un badile serve nei campi.	[P2] Un brillio è una luminosità intensa.	[P2] Un'ustione è una grave scottatura.
	[C] Il lavoro serve nei campi.	[C] La felicità è una luminosità intensa.	[C] Un tradimento è una grave scottatura.
		**Experiment 2**	
CM	[P1] Il corpo è un tempio.	[P1] Una separazione è un ristoro.	[P1] La finitura è uno sfregio.
	[P2] Un tempio è sacro.	[P2] Un ristoro ridà benessere.	[P2] Uno sfregio è una ferita.
	[C] Il corpo è sacro.	[C] Una separazione ridà benessere.	[C] La finitura è una ferita.
	[P1] Una risata è un farmaco.	[P1] La disubbidienza è la vitalità.	[P1] L'eroismo è un trauma.
	[P2] Un farmaco serve alla salute.	[P2] La vitalità è energia.	[P2] Un trauma è un evento violento.
	[C] Una risata serve alla salute.	[C] La disubbidienza è energia.	[C] L'eroismo è un evento violento.
	[P1] La vita è un capitolo.	[P1] L'indifferenza è un antidoto.	[P1] L'ammirazione è un tonfo.
	[P2] Un capitolo è una parte di un libro.	[P2] Un antidoto contrasta i veleni.	[P2] Un tonfo è una caduta a terra.
	[C] La vita è una parte di un libro.	[C] L'indifferenza contrasta i veleni.	[C] L'ammirazione è una caduta a terra.
	[P1] Quella ragazza è una gemma.	[P1] Un distacco è una schiarita.	[P1] Lo spettacolo è un ingorgo.
	[P2] Una gemma è una pietra preziosa.	[P2] Una schiarita è il ritorno del sereno.	[P2] Un ingorgo è un intasamento.
	[C] Quella ragazza è una pietra preziosa.	[C] Un distacco è il ritorno del sereno.	[C] Lo spettacolo è un intasamento.
NM	[P1] Il gallo è un faraone.	[P1] L'insolenza è una risata.	[P1] Il bagno è un accusato.
	[P2] Un faraone è un capo.	[P2] Una risata è un'espressione di gioia.	[P2] Un accusato è un possibile colpevole.
	[C] Il gallo è un capo.	[C] L'insolenza è un'espressione di gioia.	[C] Il bagno è un possibile colpevole.
	[P1] Un grido è un megafono.	[P1] Il silenzio è un alleato.	[P1] Il casinò è una slealtà.
	[P2] Un megafono emette suoni.	[P2] Un alleato è un amico.	[P2] Una slealtà è un'attività disonesta.
	[C] Un grido emette suoni.	[C] Il silenzio è un amico.	[C] Il casinò è un'attività disonesta.
	[P1] Il cuore è un'anfora.	[P1] Il dolore è un vaccino.	[P1] L'ateneo è un tugurio.
	[P2] Un'anfora trasporta liquidi.	[P2] Un vaccino rende immuni.	[P2] Un tugurio è in pessime condizioni.
	[C] Il cuore trasporta liquidi.	[C] Il dolore rende immuni.	[C] L'ateneo è in pessime condizioni.
	[P1] Il lavoro è un badile.	[P1] Una battaglia è un brillio.	[P1] La religione è un'ustione.
	[P2] Un badile serve nei campi.	[P2] Un brillio è una luminosità intensa.	[P2] Un'ustione è una grave scottatura.
	[C] Il lavoro serve nei campi.	[C] Una battaglia è una luminosità intensa.	[C] La religione è una grave scottatura.

**Table A3:** Table of literal arguments (fillers in Experiment 1 and Experiment 2).

	**Strong arguments**	**Weak arguments**	**Arguments with plausible conclusion**
Literal	[P1] Il cane rincorre il gatto.	[P1] La sarta rammenda il vestito.	[P1] Chiara ha una zia.
	[P2] Il gatto è un felino.	[P2] Il vestito è un indumento.	[P2] Una zia è la moglie dello zio.
	[C] Il cane rincorre un felino.	[C] La sarta è un indumento.	[C] Chiara è la moglie dello zio.
	[P1] Brad Pitt è una persona.	[P1] Il lunedì inizia la settimana.	[P1] Antonio richiama un soldato.
	[P2] Una persona è un essere umano.	[P2] La settimana ha sette giorni.	[P2] Un soldato è un militare.
	[C] Brad Pitt è un essere umano.	[C] Il lunedì ha sette giorni.	[C] Antonio è un militare.
	[P1] Il Louvre è un museo.	[P1] La nonna prepara la cena.	[P1] La nipote visita la zia.
	[P2] Un museo espone oggetti.	[P2] La cena è un pasto.	[P2] La zia ha sessant'anni.
	[C] Il Louvre espone oggetti.	[C] La nonna è un pasto.	[C] La nipote ha sessant'anni.
	[P1] La mucca produce il latte.	[P1] Melania mangia la mela.	[P1] Carla possiede una bottiglia.
	[P2] Il latte è un alimento.	[P2] La mela è un frutto.	[P2] Una bottiglia ha un collo.
	[C] La mucca produce un alimento.	[C] Melania è un frutto.	[C] Carla ha un collo.

## References

[B1] Angie A. D. Connelly S. Waples E. P. Kligyte V. (2011). The influence of discrete emotions on judgement and decision-making: a meta-analytic review. Cogn. Emot. 25, 1393–1422. 10.1080/02699931.2010.550751 21500048

[B2] Ariely D. (2009). Predictably Irrational. New York, NY: Harper Collins Publishers.

[B3] Ball L. G. Thompson V. A. (2018). Belief bias and reasoning, in International Handbook of Thinking and Reasoning, eds Ball L. G. Thompson V. A. (New York, NY: Routledge), 16–36.

[B4] Ball L. J. Phillips P. Wade C. N. Quayle J. D. (2006). Effects of belief and logic on syllogistic reasoning: eye-movement evidence for selective processing models. Exp. Psychol. 53, 77–86. 10.1027/1618-3169.53.1.77 16610275

[B5] Bambini V. Resta D. Grimaldi M. (2014). A dataset of metaphors from the Italian literature: exploring psycholinguistic variables and the role of context. PLoS ONE 9:e105634. 10.1371/journal.pone.0105634 25244522 PMC4171463

[B6] Barrett L. F. Mesquita B. Ochsner K. N. Gross J. J. (2007). The experience of emotion. Annu. Rev. Psychol. 58, 373–403. 10.1146/annurev.psych.58.110405.085709 17002554 PMC1934613

[B7] Barth E. M. (1974). Argument by analogy, in The Logic of the Articles in Traditional Philosophy (Springer: Dordrecht), 291–336. 10.1007/978-94-010-9866-3

[B8] Baumard N. Boyer P. (2013). Religious beliefs as reflective elaborations on intuitions: a modified dual- process model. Curr. Direct. Psychol. Sci. 22, 295–300. 10.1177/0963721413478610

[B9] Beardsley M. C. (1957). Thinking Straight. Englewood Cliffs, NJ: Prentice-Hall.

[B10] Black M. (1954). Metaphor. Proc. Aristotelian Soc. 55, 273–294. 10.1093/aristotelian/55.1.273

[B11] Black M. (1962). Models and Metaphors. Ithaca, NY: Cornell University Press. 10.7591/9781501741326

[B12] Blackburn S. (1984). Spreading the Word: Groundings in the Philosophy of Language. Oxford: Clarendon Press.

[B13] Blanchette I. (2006). The effect of emotion on interpretation and logic in a conditional reasoning task. Memory Cogn. 34, 1112–1125. 10.3758/BF03193257 17128609

[B14] Blanchette I. (2014). (eds.). Emotion and Reasoning. London; New York, NY: Psychology Press. 10.4324/9781315888538

[B15] Blanchette I. Campbell M. (2012). Reasoning about highly emotional topics: syllogistic reasoning in a group of war veterans. J. Cogn. Psychol. 24, 157–164. 10.1080/20445911.2011.603693

[B16] Blanchette I. Caparos S. (2013). When emotions improve reasoning: the possible roles of relevance and utility. Thinking Reason. 19, 399–413. 10.1080/13546783.2013.791642

[B17] Blanchette I. Caparos S. Trémolière B. (2018). Emotion and reasoning, in International Handbook of Thinking and Reasoning, eds Ball L. G. Thompson V. A. (New York, NY: Routledge), 57–70.

[B18] Blanchette I. Leese J. (2011). Physiological arousal and logicality: the effect of emotion on conditional reasoning. Exp. Psychol. 58, 235–246. 10.1027/1618-3169/a000090 21106478

[B19] Blanchette I. Richards A. (2004). Reasoning about emotional and neutral materials. Is logic affected by emotion? Psychol. Sci. 15, 745–752. 10.1111/j.0956-7976.2004.00751.x 15482446

[B20] Blanchette I. Richards A. (2010). The influence of affects on higher level cognition: a review of research on interpretation, judgement, decision making and reasoning. Cogn. Emotion 24, 561–595. 10.1080/02699930903132496

[B21] Blanchette I. Richards A. Melnyk L. Lavda A. (2007). Reasoning about emotional contents following shocking terrorist attacks: a tale of three cities. J. Exp. Psychol. Appl. 13, 47–56. 10.1037/1076-898X.13.1.47 17386001

[B22] Bless H. Clore G. L. Schwarz N. Golisano V. Rabe C. Wölk M. (1996). Mood and the use of scripts: does being in a happy mood really lead to mindlessness? J. Personal. Soc. Psychol. 71, 665–679. 10.1037/0022-3514.71.4.665 8888596

[B23] Borelli E. Cacciari C. (2019). The comprehension of metaphorical descriptions conveying gender stereotypes. An exploratory study. Front. Psychol. 10:2615. 10.3389/fpsyg.2019.02615 31824386 PMC6882934

[B24] Bradley M. M. Lang P. J. (1999). Affective Norms for English Words (ANEW): Instruction Manual and Affective Ratings (30, 1, 25–36). Technical Report C-1, the Center for Research in Psychophysiology. Gainesville, FL: University of Florida.

[B25] Burgers C. Konijn E. A. Steen G. J. (2016). Figurative framing: shaping public discourse through metaphor, hyperbole, and irony. Commun. Theory 26, 410–430. 10.1111/comt.12096

[B26] Caparos S. Blanchette I. (2015). Affect et pense logique: comment les motions influencent notre raisonnement. Revue Qubecoise de Psychologie, 36, 57–70.

[B27] Caparos S. Blanchette I. (2016). Independent effects of relevance and arousal on deductive reasoning. Cogn. Emotion 31, 1012–1022. 10.1080/02699931.2016.1179173 27144977

[B28] Carruthers P. (2011). The Opacity of Mind: An Integrative Theory of Self-Knowledge. Oxford: Oxford University Press. 10.1093/acprof:oso/9780199596195.001.0001

[B29] Carston R. (1997). Enrichment and loosening: complementary processes in deriving the proposition expressed? Linguistische Berichte 8, 103–127. 10.1007/978-3-663-11116-0_7

[B30] Carston R. (2002). Thoughts and Utterances: The Pragmatics of Explicit Communication. Oxford: Blackwell. 10.1002/9780470754603

[B31] Carston R. (2010). Metaphor: *ad hoc* concepts, literal meaning and mental images. Proc. Aristotelian Soc. 110, 295–321. 10.1111/j.1467-9264.2010.00288.x

[B32] Carston R. (2018). Figurative language, mental imagery and pragmatics. Metaphor. Symbol 33, 198–217. 10.1080/10926488.2018.1481257

[B33] Caruana F. Cuccio V. (2017). Overcoming the acting/reasoning dualism in intelligent behavior. Phenom. Cogn. Sci. 16, 709–713. 10.1007/s11097-016-9471-1

[B34] Cassotti M. Habib M. Poirel N. Aïte A. Houdé O. Moutier S. (2012). Positive emotional context eliminates the framing effect in decision-making. Emotion 12:926. 10.1037/a0026788 22309727

[B35] Cavazzana A. Bolognesi M. (2020). Uncanny resemblance: words, pictures, and conceptual representations in the field of metaphor. Cogn. Linguist. Stud. 7, 31–57. 10.1075/cogls.00048.cav

[B36] Centerbar D. B. Schnall S. Clore G. L. Garvin E. D. (2008). Affective incoherence: when affective concepts and embodied reactions clash. J. Personal. Soc. Psychol. 94, 560–578. 10.1037/0022-3514.94.4.560 18361672 PMC2365308

[B37] Channon S. Baker J. (1994). Reasoning strategies in depression: effects of depressed mood on a syllogism task. Personal. Individ. Differ. 17, 707–711. 10.1016/0191-8869(94)90148-1

[B38] Citron F. M. M. (2012). Neural correlates of written emotion word processing: a review of recent electrophysiological and hemodynamic neuroimaging studies. Brain Lang. 122, 211–226. 10.1016/j.bandl.2011.12.007 22277309

[B39] Citron F. M. M. Goldberg A. E. (2014). Metaphorical sentences are more emotionally engaging than their literal counterparts. J. Cogn. Neurosci. 26, 2585–2595. 10.1162/jocn_a_00654 24800628

[B40] Citron F. M. M. Gray M. A. Critchley H. D. Weekes B. S. Ferstl E. C. (2014). Emotional valence and arousal affect reading in an interactive way: neuroimaging evidence for an approach-withdrawal framework. Neuropsychologia 56, 79–89. 10.1016/j.neuropsychologia.2014.01.002 24440410 PMC4098114

[B41] Citron F. M. M. Weekes B. S. Ferstl E. C. (2013). Effects of valence and arousal on written word recognition: time course and ERP correlates. Neurosci. Lett. 533, 90–95. 10.1016/j.neulet.2012.10.054 23142715

[B42] Clark S. R. L. (1994). The possible truth of metaphor. Int. J. Philos. Stud. 2, 19–30. 10.1080/09672559408570781

[B43] Clore G. L. Huntsinger J. R. (2007). How emotions inform judgment and regulate thought. Trends Cogn. Sci. 11, 393–399. 10.1016/j.tics.2007.08.005 17698405 PMC2483304

[B44] Clore G. L. Schnall S. (2005). The influence of affect on attitude, in The Handbook of Attitudes, eds Albarracin D. Johnson B. T. Zanna M. P. (Mahwah, NJ: Erlbaum), 437–489.

[B45] Clore G. L. Schnall S. (2008). Affective coherence: affect as embodied evidence in attitude, advertising and art, in Embodied Grounding: Social, Cognitive, Affective, and Neuroscientific Approaches, eds Semin G. R. Smith E. R. (Cambridge: Cambridge University Press), 211–236. 10.1017/CBO9780511805837.010

[B46] Copi I. M. Cohen C. McMahon K. (2014). Introduction to Logic. Harlow: Pearson.

[B47] Correia V. (2011). Biases and fallacies: the role of motivated irrationality in fallacious reasoning. Cogency J. Reason. Argument. 3, 107–126.

[B48] Damasio A. (1994). Descartes' Error: Emotion, Reason, and the Human Brain. New York, NY: G. P. Putnam's Sons.

[B49] D'Arms J. Jacobson D. (2003). The significance of recalcitrant emotions; or anti-quasi judgmentalism. Philos. Trans. R Soc. 52, 127–146. 10.1017/CBO9780511550270.009

[B50] De Mauro T. (2000). GRADIT: Grande Dizionario Italiano Dell'uso. Torino: UTET.

[B51] De Neys W. (2012). Bias and conflict: a case for logical intuitions. Perspectiv. Psychol. Sci. 7, 28–38. 10.1177/1745691611429354 26168420

[B52] DeLancey C. (2002). Passionate Engines. What Emotions reveals about Mind and Artificial Intelligence. New York: Oxford University Press. 10.1093/0195142713.001.0001

[B53] DeLancey C. (2007). Emotion and Cognition: a new map of the terrain, in Cartographies of the Mind. Philosophy and Psychology in Intersection, eds Marraffa M. De Caro M. Ferretti F. (Berlin: Springer), 93–103. 10.1007/1-4020-5444-0_7

[B54] Dijksterhuis A. (2004). Think different: the merits of unconscious thought in preference development and decision making. J. Pers. Soc. Psychol. 87, 586–598. 10.1037/0022-3514.87.5.586 15535773

[B55] Dijksterhuis A. Bos M. W. Nordgren L. F. van Baaren R. B. (2006). On making the right choice: the deliberation-without-attention effect. Science 311, 1005–1007. 10.1126/science.1121629 16484496

[B56] Dijksterhuis A. Bos M. W. Van Der Leij A. Van Baaren R. B. (2009). Predicting soccer matches after unconscious and conscious thought as a function of expertise. Psychol. Sci. 20, 1381–1387. 10.1111/j.1467-9280.2009.02451.x 19818044

[B57] Elbow P. (1998). The Doubting Game and the Believing Game. An Analysis of the Intellectual Enterprise. In Writing Without Teachers. New York, NY: Oxford University Press.

[B58] Eliades M. Mansell W. Blanchette I. (2013). The effect of emotion on statistical reasoning: findings from a base rates task. J. Cogn. Psychol. 25, 277–282. 10.1080/20445911.2012.761632

[B59] Eliades M. Mansell W. Stewart A. J. Blanchette I. (2012). An investigation of belief-bias and logicality in reasoning with emotional contents. Thinking Reason. 18, 461–479. 10.1080/13546783.2012.713317

[B60] Elster J. (1999). Alchemies of the Mind: Rationality and the Emotions. New York, NY: Cambridge University Press. 10.1017/CBO9781139173308

[B61] Entman R. M. (1993). Framing: toward clarification of a fractured paradigm. J. Commun. 43, 51–58. 10.1111/j.1460-2466.1993.tb01304.x

[B62] Ervas F. (2015). (Becoming) experts in meaning ambiguities. Humana. Mente. J. Philos. Stud. 8, 225–243.

[B63] Ervas F. (2017). Another metaphor is possible. Challenging social stereotypes in figurative language comprehension. Reti Saperi Linguaggi 4, 79–96. 10.12832/87357

[B64] Ervas F. (2019). Metaphor, ignorance and the sentiment of (ir) rationality. Synthese 19, 1–25. 10.1007/s11229-019-02489-y

[B65] Ervas F. (2020). How nice does it sound? An argumentative approach to the affective aspects of irony, in Producing Figurative Expression: Theoretical, Experimental and Practical Perspectives, eds Barnden J. Gargett A. (Amsterdam: John Benjamins), 175–210. 10.1075/ftl.10.07erv

[B66] Ervas F. Gola E. Rossi M. G. (2015). Metaphors and emotions as framing strategies in argumentation. Proc. EuroAsianPacific Joint Conf. Cogn. Sci. 1419, 645–650.

[B67] Ervas F. Gola E. Rossi M. G. (2018). Argumentation as a bridge between metaphor and reasoning, in Argumentation and Language. Linguistic, Cognitive and Discursive Explorations, eds Oswald S. Herman T. Jacquin J. (Berlin: Springer), 153–170. 10.1007/978-3-319-73972-4_7

[B68] Evans J. (2008). Dual-processing accounts of reasoning, judgment and social cognition. Ann. Rev. Psychol. 59, 255–278. 10.1146/annurev.psych.59.103006.093629 18154502

[B69] Evans J. Barston J. L. Pollard P. (1983). On the conflict between logic and belief in syllogistic reasoning. Mem. Cogn. 11, 295–306. 10.3758/BF03196976 6621345

[B70] Evans J. Frankish K. (2009). In Two Minds. Dual Processes and Beyond. Oxford: Oxford University Press. 10.1093/acprof:oso/9780199230167.001.0001

[B71] Fainsilber L. Ortony A. (1987). Metaphorical uses of language in the expression of emotions. Metaphor Symb. Act. 2, 239–250. 10.1207/s15327868ms0204_2

[B72] Fischer E. (2014). Philosophical intuitions, heuristics, and metaphors. Synthese 191, 569–606. 10.1007/s11229-013-0292-2

[B73] Fischer E. (2015). Mind the metaphor! A systematic fallacy in analogical reasoning. Analysis 75, 67–77. 10.1093/analys/anu124

[B74] Fletcher L. Carruthers P. (2012). Metacognition and reasoning. Philos. Trans. R Soc. Lond. B Biol. Sci. 367, 1366–1378. 10.1098/rstb.2011.0413 22492753 PMC3318763

[B75] Forgas J. P. (1995). Mood and judgment: the affect infusion model. Psychol. Bulletin 117, 39–66. 10.1037/0033-2909.117.1.39 7870863

[B76] Fredrickson B. L. Branigan C. (2005). Positive emotions broaden the scope of attention and thought-action repertoires. Cogn. Emotion 19, 313–332. 10.1080/02699930441000238 21852891 PMC3156609

[B77] Gernsbacher M. A. Faust M. (1991). The role of suppression in sentence comprehension, in Understanding Word and Sentence, ed Simpson G. B. (Amsterdam: Elsevier), 97–128. 10.1016/S0166-4115(08)61531-9

[B78] Gernsbacher M. A. Keysar B. Robertson R. R. W. Werner N. K. (2001). The role of suppression and enhancement in understanding metaphors. J. Mem. Lang. 45, 433–450. 10.1006/jmla.2000.2782 25110388 PMC4123822

[B79] Gibbs R. W. Leggitt J. S. Turner E. A. (2002). What's special about figurative language in emotional communication, in The Verbal Communication of Emotions: Interdisciplinary Perspectives, ed Fussell S. R. (Mahwah, NJ: Lawrence Erlbaum Associates), 125–149.

[B80] Gibbs R. W. Jr. (2006). Metaphor interpretation as embodied simulation. Mind Language 21, 434–458. 10.1111/j.1468-0017.2006.00285.x

[B81] Giora R. (2003). On Our Mind: Salience, Context and Figurative Language. Oxford: Oxford University Press. 10.1093/acprof:oso/9780195136166.001.0001

[B82] Glucksberg S. (2001). Understanding Figurative Language: From Metaphors to Idioms. Oxford: Oxford University Press. 10.1093/acprof:oso/9780195111095.001.0001

[B83] Glucksberg S. (2003). The psycholinguistics of metaphor. Trends Cogn. Sci. 7, 92–96. 10.1016/S1364-6613(02)00040-2 12584028

[B84] Glucksberg S. Gildea P. Bookin H. B. (1982). On understanding nonliteral speech: can people ignore metaphors? J. Verbal Learning Verbal Behav. 21, 85–98. 10.1016/S0022-5371(82)90467-4

[B85] Godden D. (2015). Argumentation, rationality, and psychology of reasoning. Inf. Logic 35, 135–166. 10.22329/il.v35i2.4124

[B86] Goel V. Vartanian O. (2011). Negative emotions can attenuate the influence of beliefs on logical reasoning. Cogn. Emotion 25, 121–131. 10.1080/02699931003593942 21432659

[B87] Greenspan P. S. (1988). Emotions and Reasons: An Inquiry Into Emotional Justification. New York, NY: Routledge, Chapman and Hall.

[B88] Greenspan P. S. (1992). Subjective guilt and responsibility. Mind 101, 287–303. 10.1093/mind/101.402.287

[B89] Greenspan P. S. (2004). Emotions, rationality, and mind-body, in Thinking About Feeling: Contemporary Philosophers on Emotions, ed Solomon R. C. (New York, NY: Oxford University Press), 125–134.

[B90] Grice P. (1975). Logic and conversation, in Syntax and Semantics 3: Speech Acts, eds Cole P. Morgan J. (New York, NY: Academic Press), 41–58. 10.1163/9789004368811_003

[B91] Grice P. (1989). Studies in the Ways of Words. Cambridge, MA: Harvard University Press.

[B92] Gross J. J. (2008). Emotion regulation, in Handbook of Emotions, eds Lewis M. Haviland-Jones J. M. Barrett L. F. (New York, NY: The Guilford Press), 497–512.

[B93] Gross K. D'Ambrosio L. (2004). Framing emotional response. Polit. Psychol. 25, 1–29. 10.1111/j.1467-9221.2004.00354.x

[B94] Haack S. (1994). “‘Dry truth and real knowledge”': epistemologies of metaphor and metaphors of epistemology,” in Aspects of Metaphor. Synthese Library, vol 238, ed Hintikka J. (Dordrecht: Springer), 1–22.

[B95] Haidt J. (2001). The emotional dog and its rational tail: a social intuitionist approach to moral judgment. Psychol. Rev. 108, 814–834. 10.1037/0033-295X.108.4.814 11699120

[B96] Haidt J. (2007). The new synthesis in moral psychology. Science 316, 998–1002. 10.1126/science.1137651 17510357

[B97] Haidt J. Bjorklund F. Murphy S. (2000). Moral dumbfounding: when intuition finds no reason. Lund Psychol. Rep. 1, 191–221.

[B98] Hamblin C. L. (1970). Fallacies. London: Methuen.

[B99] Hesse M. (1963). Models and Analogies in Science. London: Sheed and Ward.

[B100] Hesse M. (1965). Aristotle's logic of analogy. Philos. Q. 15, 328–340. 10.2307/2218258

[B101] Hinton M. (2019). Language and argument: a review of the field. Res. Lang. 17, 93–103. 10.2478/rela-2019-0007

[B102] Hoffman R. R. (1980). Metaphor in science, in The Psycholinguistics of Figurative Language, eds Honeck R. P. Hoffman R. R. (Hillsdale, NJ: Erlbaum), 393–423. 10.4324/9780429432866-16

[B103] Hofmann S. G. Heering S. Sawyer A. T. Asnaani A. (2009). How to handle anxiety: the effects of reappraisal, acceptance, and suppression strategies on anxious arousal. Behav. Res. Therapy 47, 389–394. 10.1016/j.brat.2009.02.010 19281966 PMC2674518

[B104] Hofstadter D. (1995). Fluid Concepts and Creative Analogies: Computer Models of the Fundamental Mechanisms of Thought. New York, NY: Basic Books.

[B105] Huntsinger J. R. (2013). Incidental experiences of affective coherence and incoherence influence persuasion. Pers. Soc. Psychol. Bull. 39, 792–802. 10.1177/0146167213482588 23542415

[B106] Indurkhya B. (1992). Metaphor and Cognition. Dordrecht: Kluwer Academic Publishers. 10.1007/978-94-017-2252-0

[B107] Indurkhya B. (1994). Metaphor as change of representation: an interaction theory of cognition and metaphor, in Aspects of Metaphor. Synthese Library, ed Hintikka J. (Dordrecht: Springer), 95–150.

[B108] Indurkhya B. (2006). Emergent representations, interaction theory and the cognitive force of metaphor. New Ideas Psychol. 24, 133–162. 10.1016/j.newideapsych.2006.07.004

[B109] Indurkhya B. (2007). Rationality and reasoning with metaphors. New Ideas Psychol. 25, 16–36. 10.1016/j.newideapsych.2006.10.006

[B110] Indurkhya B. (2010). On the role of metaphor in creative cognition, in Proceedings of the International Conference on Computational Creativity: ICCC-X, eds Ventura D. Pease A. Perez y Perez R. Ritchie G. Veale T. (Department of Informatics Engineering, University of Coimbra: Coimbra), 51–59.

[B111] Indurkhya B. (2016). Towards a model of metaphorical understanding, in Metaphor and Communication, eds Gola E. Ervas F. (Amsterdam: John Benjamins), 123–146. 10.1075/milcc.5.07ind

[B112] Isen A. M. Daubman K. A. Nowicki G. P. (1987). Positive affect facilitates creative problem solving. J. Pers. Soc. Psychol. 52, 1122–1131. 10.1037/0022-3514.52.6.1122 3598858

[B113] Johnson-Laird P. N. Mancini F. Gangemi A. (2006). A hyper-emotion theory of psychological illnesses. Psychol. Rev. 113, 822–841. 10.1037/0033-295X.113.4.822 17014304

[B114] Jung N. Wranke C. Hamburger K. Knauff M. (2014). How emotions affect logical reasoning: evidence from experiments with mood-manipulated participants, spider phobics, and people with exam anxiety. Front. Psychol. 5:570. 10.3389/fpsyg.2014.00570 24959160 PMC4050437

[B115] Kahneman D. (2003). A perspective on judgment and choice: mapping bounded rationality. Am. Psychol. 58, 697–720. 10.1037/0003-066X.58.9.697 14584987

[B116] Keefer L. A. Landau M. J. (2016). Metaphor and analogy in everyday problem solving. Wiley Interdiscip. Rev. Cogn. Sci. 7, 394–405. 10.1002/wcs.1407 27582354

[B117] Kensinger E. A. Corkin S. (2003). Effect of negative emotional content on working memory and long-term memory. Emotion 3, 378–393. 10.1037/1528-3542.3.4.378 14674830

[B118] Kissler J. Assadollahi R. Herbert C. (2006). Emotional and semantic networks in visual word processing: insights from ERP studies. Prog. Brain Res. 156, 147–183. 10.1016/S0079-6123(06)56008-X 17015079

[B119] Kousta S.-T. Vinson D. P. Vigliocco G. (2009). Emotion words, regardless of polarity, have a processing advantage over neutral words. Cognition 112, 473–481. 10.1016/j.cognition.2009.06.007 19591976

[B120] Kövecses Z. (2000). Metaphor and Emotions. Cambridge: Cambridge University Press.

[B121] Kövecses Z. (2005). Metaphor in Culture: Universality and Variation. Cambridge: Cambridge University Press. 10.1017/CBO9780511614408

[B122] Lakoff G. Johnson M. (1980). Metaphors We Live By. Chicago, IL: University of Chicago Press.

[B123] Larsen R. J. Mercer K. A. Balota D. A. (2006). Lexical characteristics of words used in emotional Stroop experiments. Emotion 6, 62–72. 10.1037/1528-3542.6.1.62 16637750

[B124] Lecheler S. Schuck A. R. T. de Vreese C. H. (2013). Dealing with feelings: positive and negative discrete emotions as mediators of news framing effects. Communications 38, 189–209. 10.1515/commun-2013-0011

[B125] LeDoux J. E. (1996). The Emotional Brain. The Mysterious Underpinnings of Emotional Life. New York, NY: Simon and Schuster.

[B126] Lefford A. (1946). The influence of emotional subject matter on logical reasoning. J. General Psychol. 34, 127–151. 10.1080/00221309.1946.10544530 20982983

[B127] Leung A. K. Kim S. Polman E. Ong L. Qiu L. Goncalo J. A.. (2012). Embodied metaphors and creative “acts”. Psychol. Sci. 23, 502–509. 10.1177/0956797611429801 22477105

[B128] Lieberman H. R. Bathalon G. P. Falco C. M. Morgan C. A. Niro P. J. Tharion W.J.. (2005).The fog of war: decrements in cognitive performance and mood associated with combat-like stress. Aviat. Space Environ. Med. 76, 7–14. 16018323

[B129] Lightbody B. A. Berman M. P. (2010). The metaphoric fallacy to a deductive inference. Informal Logic 30, 185–193. 10.22329/il.v30i2.1192

[B130] Maasen S. (2000). Metaphors in the social sciences: making use and making sense of them, in Metaphor and Analogy in the Sciences, ed Hallyn F. (Dordrecht: Springer), 99–244.

[B131] Macagno F. (2020). How can metaphors communicate arguments? Intercult. Pragmat. 17, 335–363. 10.1515/ip-2020-3004

[B132] Macagno F. Rossi M. G. (2021). Inferential patterns of emotive meaning. Perspect. Pragmatics Philos. Psychol. 28, 83–110. 10.1007/978-3-030-56696-8_5

[B133] Macagno F. Walton D. (2009). Argument from analogy in law, the classical tradition, and recent theories. Philos. Rhetor. 42, 154–182. 10.1353/par.0.0034

[B134] Macagno F. Walton D. (2010). The argumentative uses of emotive language. Rev. Iberoam. Argument. 1, 1–33.

[B135] Macagno F. Walton D. (2014). Emotive Language in Argumentation. Cambridge: Cambridge University Press. 10.1017/CBO9781139565776

[B136] Macagno F. Zavatta B. (2014). Reconstructing metaphorical meaning. Argumentation 28, 453–488. 10.1007/s10503-014-9329-z

[B137] MacKuen M. Wolak J. Keele L. Marcus G. E. (2010). Civic engagements: resolute partisanship or reflective deliberation. Am. J. Pol. Sci. 54, 440–458. 10.1111/j.1540-5907.2010.00440.x

[B138] Maiese M. (2014). How can emotions be both cognitive and bodily? Phenomenol. Cogn. Sci. 13, 513–531. 10.1007/s11097-014-9373-z

[B139] Maiese M. (2015). Thought insertion as a disownership symptom. Phenomenol. Cogn. Sci. 14, 911–927. 10.1007/s11097-014-9387-6

[B140] Marcus G. E. (2002). The Sentimental Citizen. Emotion in Democratic Politics. Pennsylvania, PA: The Pennsylvania State University Press.

[B141] Marcus G. E. Neuman W. R. Mackuen M. (2000). Affective Intelligence and Political Judgment. Chicago/London: The University of Chicago Press.

[B142] Marraffa M. (2014). Emozioni e razionalit: oltre il modello ≪antagonistico≫. Sistemi Intelligenti 1, 149–160. 10.1422/77223

[B143] Melton R. J. (1995). The role of positive affect in syllogism performance. Pers. Soc. Psychol. Bull. 21, 788–794. 10.1177/0146167295218001

[B144] Mercier H. Sperber D. (2011). Why do Humans reason? Arguments for an argumentative theory. Behav. Brian Sci. 34, 57–111. 10.1017/S0140525X10000968 21447233

[B145] Moshman D. (2004). From inference to reasoning: the construction of rationality. Thinking Reason. 10, 221–239. 10.1080/13546780442000024

[B146] Oakhill J. Garnham A. (1993). On theories of belief bias in syllogistic reasoning. Cognition 46, 87–92. 10.1016/0010-0277(93)90023-O 8432091

[B147] Oakhill J. Johnson-Laird P. N. Garnham A. (1989). Believability and syllogistic reasoning. Cognition 31, 117–140. 10.1016/0010-0277(89)90020-6 2721132

[B148] Oaksford M. Morris F. Grainger B. Williams J. M. G. (1996). Mood, reasoning, and central executive processes. J. Exp. Psychol. Learn. Mem. Cogn. 22, 476–492. 10.1037/0278-7393.22.2.476

[B149] Oswald S. Greco S. Miecznikowski J. Pollaroli C. Rocci A. (2020). Argumentation and meaning. Semantic and pragmatic reflexions. J. Argument. Context 9:5. 10.1075/jaic.00005.osw

[B150] Oswald S. Herman T. Jacquin J. (2018). Argumentation and Language – Linguistic, Cognitive and Discursive Explorations. Cham: Springer. 10.1007/978-3-319-73972-4

[B151] Oswald S. Rihs A. (2014). Metaphor as argument: rhetorical and epistemic advantages of extended metaphors. Argumentation 28, 133–159. 10.1007/s10503-013-9304-0

[B152] Perelman C. Olbrechts-Tyteca L. (1969). The New Rhetoric: A Treatise on Argumentation. Notre Dame: University of Notre Dame Press.

[B153] Petty R. Briñol P. (2015). Emotion and persuasion: cognitive and meta-cognitive processes impact attitudes. Cogn. Emotion 29, 1–26, 10.1080/02699931.2014.967183 25302943

[B154] Petty R. Cacioppo J. (1986a). The elaboration likelihood model of persuasion. Adv. Exp. Soc. Psychol. 19, 123–205. 10.1016/S0065-2601(08)60214-2

[B155] Petty R. Cacioppo J. (1986b). Communication and Persuasion: Central and Peripheral Routes to Attitude Change. New York, NY: Springer-Verlag.

[B156] Pollaroli C. Greco S. Oswald S. Miecznikowski J. Rocci A. (2019). Rhetoric and Language: emotions and style in argumentative discourse. Informal Logic 39:6047. 10.22329/il.v39i4.6047

[B157] Prinz J. J. (2004). Gut Reactions: A Perceptual Theory of Emotion. New York, NY: Oxford University Press.

[B158] Recanati F. (2004). Literal Meaning. Cambridge: Cambridge University Press. 10.1017/CBO9780511615382

[B159] Recanati F. (2010). Truth-Conditional Pragmatics. Oxford: Oxford University Press. 10.1093/acprof:oso/9780199226993.001.0001

[B160] Richards J. M. Gross J. L. (1999). Composure at any cost? The cognitive consequences of emotion suppression. Personal. Soc. Psychol. Bullet. 8, 1033–1044. 10.1177/01461672992511010

[B161] Robins S. Mayer R. E. (2000). The metaphor framing effect: metaphorical reasoning about text-based dilemmas. Discourse Processes 30, 57–86. 10.1207/S15326950dp3001_03

[B162] Rossi M. G. (2013). Il Giudizio del Sentimento. Emozioni, giudizi morali, natura umana. Rome: Editori Riuniti.

[B163] Rossi M. G. (2014). Emozioni e deliberazione razionale. Sistemi Intelligenti 26, 161–170. 10.1422/77224

[B164] Rozin P. Royzman E. B. (2001). Negativity bias, negativity dominance, and contagion. Personal. Soc. Psychol. Rev. 5, 296–320. 10.1207/S15327957PSPR0504_2

[B165] Rubio Fernandez P. (2007). Suppression in metaphor interpretation: differences between meaning selection and meaning construction. J. Semant. 24, 345–371. 10.1093/jos/ffm006

[B166] Santibáñez C. (2010). Metaphors and argumentation: the case of Chilean parliamentarian media participation. J. Pragmat. 42, 973–989. 10.1016/j.pragma.2009.08.019

[B167] Schauenburg G. Conrad M. von Scheve C. Barber H. A. Ambrasat J. Aryani A.. (2019). Making sense of social interaction: emotional coherence drives semantic integration as assessed by event-related potentials. Neuropsychologia 4, 1–13. 10.1016/j.neuropsychologia.2019.01.002 30664854

[B168] Scherer A. M. Scherer L. D. Fagerlin A. (2014). Getting ahead of illness: using metaphors to influence medical decision making. Med. Decis. Mak. 35, 37–45. 10.1177/0272989X14522547 24615273

[B169] Schn D. A. (1993). Generative metaphor: a perspective on problem-setting in social policy, in Metaphor and Thought, ed Orthony A. (Cambridge: Cambridge University Press), 137–163. 10.1017/CBO9781139173865.011

[B170] Schnall S. (2005). The pragmatics of emotion language. Psychol. Inq. 16, 28–31.

[B171] Schumann J. Zufferey S. Oswald S. (2020). The linguistic formulation of fallacies matters: the case of causal connectives. Argumentation 2020, 1–28. 10.1007/s10503-020-09540-0

[B172] Schwarz N. Clore G. L. (1983). Mood, misattribution, and judgments of well-being: informative and directive functions of affective states. J. Personal. Soc. Psychol. 45, 513–523. 10.1037/0022-3514.45.3.513

[B173] Schwarz N. Skurnik I. (2003). Feeling and thinking: implications for problem solving, in The Psychology of Problem Solving, eds Davidson J. E. Sternberg R. J. (Cambridge: Cambridge University Press), 263–290. 10.1017/CBO9780511615771.010

[B174] Semino E. (2008). Metaphor in discourse. Cambridge: Cambridge University Press.

[B175] Slothuus R. (2008). More than weighting cognitive importance: a dual-process model of issue framing effects. Polit. Psychol. 29, 1–28. 10.1111/j.1467-9221.2007.00610.x

[B176] Solomon R. C. (Ed.). (2003). What Is an Emotion? Oxford: Oxford University Press.

[B177] Sperber D. Wilson D. (1986). Relevance: Communication and Cognition. Oxford: Blackwell.

[B178] Steen G. J. Reijnierse W. G. Burgers C. (2014). When do natural language metaphors influence reasoning? A follow-up study to Thibodeau and Boroditsky (2013). PLoS ONE 9:e113536. 10.1371/journal.pone.0113536 25490704 PMC4260786

[B179] Stevenson C. (1944). Ethics and Language. New Haven, CT: Yale University Press.

[B180] Stocker M. Hegeman E. (1996). Valuing Emotions. Cambridge: Cambridge University Press.

[B181] Svačinova I. (2014). Reconstruction of metaphors in argumentation: a case study of Lincoln's metaphor of “swapping horses while crossing a stream”. Cogency 6, 67–89.

[B182] Tamir M. Robinson M. D. Clore G. L. (2002). The epistemic benefits of trait-consistent mood states: an analysis of extraversion and mood. J. Personal. Soc. Psychol. 83, 663–677. 10.1037/0022-3514.83.3.663 12219861

[B183] Thibodeau P. H. Boroditsky L. (2011). Metaphors we think with: the role of metaphor in reasoning. PLoS ONE 6:e16782. 10.1371/journal.pone.0016782 21373643 PMC3044156

[B184] Thibodeau P. H. Boroditsky L. (2015). Measuring effects of metaphor in a dynamic opinion landscape. PLoS ONE 10, 1–22. 10.1371/journal.pone.0133939 26218229 PMC4517745

[B185] Thibodeau P. H. Matlock T. Flusberg S. J. (2019). The role of metaphor in communication and thought. Lang. Linguist. Compass 13:e12327. 10.1111/lnc3.12327

[B186] Tindale C. W. (2006). Fallacies and Argument Appraisal. Cambridge: Cambridge University Press. 10.1017/CBO9780511806544

[B187] Tourangeau R. Sternberg R. J. (1982). Understanding and appreciating metaphors. Cognition 11, 203–244. 10.1016/0010-0277(82)90016-6 7199412

[B188] Tversky A. Kahneman D. (1981). The framing of decisions and the psychology of choice. Science 211, 453–458. 10.1126/science.7455683 7455683

[B189] Van Eemeren F. H. (1992). Argumentation, Communication and Fallacies. Hillsdale: Erlbaum.

[B190] Van Eemeren F. H. Grootendorst R. (2004). A Systematic Theory of Argumentation. The Pragma-Dialectical Approach. Cambridge: Cambridge University Press. 10.1017/CBO9780511616389

[B191] van Poppel L. (2018). Argumentative functions of metaphors: how can metaphors trigger resistance?, in Argument and Inference. Proceedings of the 2nd European Conference on Argumentation volume II, eds Oswald S. Maillat D. Fribourg.

[B192] van Poppel L. (2020). The study of metaphor in argumentation theory. Argumentation 20:9523. 10.1007/s10503-020-09523-1

[B193] Wagemans J. H. M. (2016). Analyzing metaphor in argumentative discourse. RIFL 102, 79–94. 10.4396/20161207

[B194] Wagemans J. H. M. (2019). Four basic argument forms. Res. Lang. 17, 57–69. 10.2478/rela-2019-0005

[B195] Walton D. N. (1996). Fallacies Arising from Ambiguity. Dordrecht: Springer. 10.1007/978-94-015-8632-0

[B196] Walton D. N. (2006). Fundamentals of Critical Argumentation. Cambridge, MA: Cambridge University Press.

[B197] Walton D. N. (2010). Why fallacies appear to be better arguments than they are. Inf. Log. 30, 159–184. 10.22329/il.v30i2.2868

[B198] Wilson D. Sperber D. (2002). Truthfulness and relevance. Mind 111, 583–632. 10.1093/mind/111.443.583

[B199] Woods J. Walton D. N. (1989). Fallacies: Selected Papers, 1972–1982. Dordrecht: Foris. 10.1515/9783110816082

[B200] Zajonc R. B. (1980). Feeling and thinking: preferences need no inferences. Am. Psychol. 35, 151–175. 10.1037/0003-066X.35.2.151

